# Selective and sensitive CQD-based sensing platform for Cu^2+^ detection in Wilson’s disease

**DOI:** 10.1038/s41598-024-63771-9

**Published:** 2024-06-08

**Authors:** Armin Zarei, Aram Rezaei, Mohsen Shahlaei, Zhaleh Asani, Ali Ramazani, Chuanyi Wang

**Affiliations:** 1 The Organic Chemistry Research Laboratory (OCRL), Department of Chemistry, University of Zanjan, Zanjan, 45371-38791 Iran; 2 Nano Drug Delivery Research Center, Health Technology Institute, Kermanshah University of Medical Sciences, Kermanshah, Iran; 3 Students Research Committee,, Kermanshah University of Medical Sciences, Kermanshah, Iran; 4 School of Environmental Science and Engineering, Shaanxi University of Science and Technology, Xi’an, 710021 People’s Republic of China; 5 The Convergent Sciences & Technologies Laboratory (CSTL), Research Institute of Modern Biological Techniques (RIMBT), University of Zanjan, Zanjan 45371-38791, Iran; 6 Radiology Department, Kermanshah University of Medical Sciences, Kermanshah, Iran

**Keywords:** Wilson’s disease, Carbon quantum dots, Fluorescent sensor, Cu(II) detection, Turn-on and turn-off states mechanism, Chemistry, Nanoscience and technology

## Abstract

Excessive Cu^2+^ intake can cause neurological disorders (e.g. Wilson’s disease) and adversely affect the gastrointestinal, liver, and kidney organs. The presence of Cu^2+^ is strongly linked to the emergence and progression of Wilson's disease (WD), and accurately measuring the amount of copper is a crucial step in diagnosing WD at an early stage in a clinical setting. In this work, CQDs were fabricated through a facile technique as a novel fluorescence-based sensing platform for detecting Cu(II) in aqueous solutions, and in the serum samples of healthy and affected individuals by WD. The CQDs interact with Cu(II) ions to produce Turn-on and Turn-off states at nano-molar and micro-molar levels, respectively, with LODs of 0.001 µM and 1 µM. In fact, the Cu^2+^ ions can act like a bridge between two CQDs by which the charge and electron transfer between the CQDs may increase, possibly can have significant effects on the spectroscopic features of the CQDs. To the best of our knowledge, this is the first reported research that can detect Cu(II) at low levels using two different complexation states, with promising results in testing serum. The potential of the sensor to detect Cu(II) was tested on serum samples from healthy and affected individuals by WD, and compared to results obtained by ICP-OES. Astonishingly, the results showed an excellent correlation between the measured Cu(II) levels using the proposed technique and ICP-OES, indicating the high potential of the fluorimetric CQD-based probe for Cu(II) detection. The accuracy, sensitivity, selectivity, high precision, accuracy, and applicability of the probe toward Cu(II) ions make it a potential diagnostic tool for Wilson's disease in a clinical setting.

## Introduction

Copper is a vital trace element and cofactor for approximately 20 enzymes, crucial for metabolic functions and various biological processes^1–3^. Free copper ions (Cu^2+^) aid bone production, cellular respiration, and connective tissue development^4,5^. However, excessive Cu^2+^ intake can harm the gastrointestinal system, liver, kidneys organs,^6^ and cause neurological disorders such as Alzheimer's, Parkinson's, and Wilson’s disease^7–9^. Wilson's disease (WD) is a genetic disorder caused by mutations in the ATP7B gene, disrupting copper transport^10^, leads to copper accumulation in the brain, liver, and kidneys. In advanced stages of WD, liver cirrhosis and failure can occur, potentially leading to death^11,12^. Thus, developing high-performance techniques for early copper detection is crucial for WD diagnosis, symptom alleviation, and extending patients' lifespans^13^. Typically, serum copper ranges from 15.7 to 23.6 µM, while it is believed that the level is lower in WD patients due to copper accumulation in the liver^14^. Therefore, developing straightforward, fast, precise, and reliable techniques for selective/sensitive identification and measurement of copper in biological and environmental samples is crucial.

Conventional methods such as ICP-MS (inductively coupled plasma mass spectroscopy)^12,15^ and AAS (atomic absorption spectrometry)^16,17^ are currently used for clinical copper detection. Despite their sensitivity and selectivity, these techniques are costly and may be inefficient for widespread metal detection, they suffer from some demerits such as expensive instruments and high operation costs, which might diminish their efficiency for metal detection^18^. Some other techniques have been reported for serum copper detection in WD such as photoacoustic Imaging^9^, fluorescence imaging^19^, colorimetry^20^, fluorimetry, and so on^8,21–25^. In 2018, Li et al. have developed a colorimetric cyanine probe for the Cu^2+^ detection in WD, which showed a detection limit of 0.08 µM^20^. In 2021, Song and co-workers have introduced a new sensing platform relying on Au NPs and poly(N-isopropylacrylamide) for the determination of copper ions trough photoacoustic Imaging in urinary samples in WD^9^. This probe exhibited a limit of detection (LOD) of 40.8 µM^9^.

Compared to the Cu^2+^-detection techniques as mentioned earlier, the fluorescence method has gained tremendous enthusiasm due to its beneficial advantages such as improved sensitivity, low cost, user-friendliness, and simplicity^26,27^. To date, various sensing probes have been presented to detect copper ions in WD. For example, Taghdisi et al. have produced a DNA aptamer-based sensor for Cu^2+^ detection in serum samples, which showed a detection limit of 0.01 μM^8^. In another job, Eu-MOF sensing platform was rationally fabricated to detect Cu^2+^ in serum samples for the diagnosis of WD, shown a LOD of 0.15 µM^21^. Among the fluorimetric-based probe for copper ions detection, carbon quantum dot (CQD)-based probes have aroused much attention.

Over the last decade, CQDs have become popular due to their small size, low toxicity, steady chemical inertness, stable fluorescence, and high quantum yield^28–42^. These properties make CQDs ideal for sensitive sensing applications^43–45^. In recent years, fluorescence-based sensors using CQDs have been developed to detect copper ions by quenching or boosting fluorescence. In 2019, a CQD-based "turn-off" sensor for Cu^2+^ detection was prepared from Finger millet ragi with a LOD of 0.01 µM^46^. Another developed probe could detect Cu^2+^ with a 0.004 µM LOD, as reported by Bhamore and co-workers^47^. However, some CQD-based sensors for copper detection are either based on "turn-on" fluorescence or dual emission emitter probes For example, a sensor developed from citric acid and o-phenylenediamine detected copper ions and glutathione with LODs of 76 µM and 0.003 µM, respectively^48^. Additionally, paper-based platforms offer low-cost, precise sensing, enabling quick^49–51^, on-site, visual, and quantitative analysis for food safety and environmental protection.

Although most previously developed nano-sensors use turn-off or turn-on fluorescence, but rarely CQD-based probes detect Cu^2+^ at two concentrations with linear response from nano- to micro-molar levels. This study presents a simple, effective CQD-based fluorimetric strategy for sensitive and selective Cu^2+^ detection, using the one-pot synthesis method with neocuproine and citric acid in an autoclave. The as-prepared CQDs were fully characterized using different spectroscopic and electron microscopic analyses. To the best of our knowledge, this is the first reported nano-sensor for Cu^2+^ determination not only in nano-molar, but also in micro-molar levels in which CQDs FL emission experienced two distinguishable phenomena. Amazingly, at trace amounts of 0.001 µM, Cu^2+^ enhances the CQDs’ FL emission (Turn-on) with a R^2^ of 0.9993, while at higher concentrations (1–10 µM), it quenches the emission (Turn-off) with a R^2^ of 0.9886. This dual response led to the development of a paper-based fluorescence probe for visual Cu^2+^ detection. The probe effectively measured copper in the serum of healthy individuals and Wilson's disease patients, with results comparable to ICP-OES. The detection mechanism was explored using fluorescence, ζ-potential, and DLS analysis, supported by DFT calculations to understand the nature of complexations between Cu^2+^ ions and CQDs.

## Materials and methods

### Chemicals and apparatus

All commercially available chemicals such as phosphoric acid (H_3_PO_4_), calcium chloride (CaCl_2_), aluminium nitrate (Al(NO_3_)_3_), potassium chloride (KCl), iron(II) chloride (FeCl_2_), ferric chloride (FeCl_3_), sodium sulfate (Na_2_SO_4_), copper(II) chloride (CuCl_2_), mercuric chloride (HgCl_2_), lead(II) chloride (PbCl_2_), silver chloride (AgCl_2_), zinc chloride (ZnCl_2_), lithium hydroxide (LiOH), ammonium hydroxide (NH_4_OH), cobalt chloride (CoCl_2_), chromium oxide (Cr_2_O_3_), nickel(II) hydroxide (Ni(OH)_2_), magnesium chloride (MgCl_2_), manganese(II) chloride (MnCl_2_), citric acid, and neocuproine were purchased from Sigma-Aldrich and Merck (Darmstadt, Germany) and applied as received. Deionized water was applied in all of the experiments. The ^1^H NMR and ^13^C NMR spectra were taken on a Bruker Avance DPX 300 (300 MHz) NMR spectrometer (Ettlingen, Germany) via tetramethylsilane (TMS) as an internal standard. The FT-IR spectra were taken from 400 to 4000 cm^-1^ through the KBr pellet approach via Perkin-Elmer IR spectrometer. The optical properties of CQDs was determined by a UV visible spectrophotometer (Shimadzu UV 2100 PC UV visible spectrophotometer, Kyoto, Japan). The XPS measurements were conducted by an ESCALab MKII spectrometer (Thermo Fisher Scientific) and Al Kα (hν = 1.4866 keV) as an X-ray source. The shift of the binding energy was corrected using the C 1 s level (at 284.8 eV) as an internal standard. TGA (Thermal gravimetric analysis) was supplemented via a TGA Q 50 thermogravimetric analyzer at a heating rate of 10 °C min^-1^ under N_2_ flow. The fluorescence spectra were taken through a spectrofluorometer (Perkin-Elmer) with an excitation wavelength of 300 nm at ambient temperature. The amount of Cu^2+^ ions in the plasma samples have been measured by ICP-OES as standard by ICP-OES SPECTRO ARCOS. The DLS and Zeta potential have been obtained by DLS Horiba.

### Synthesis of the CQDs

The CQDs were fabricated through a mild condition according to earlier works^52,53^. Shortly, neocuproine and citric acid (1:2 molar ratio) were initially mixed in 15 mL beacher containing DI water to be sonicated for 15 min before transferring the gained solution into a 50-mL Teflon-lined stainless-steel autoclave and heated at a steady temperature of 180 ◦C for 5 h, CQDs were gained as a colorless dispersion. The solution was then cooled down to room temperature and centrifuged for 15 min before the collection of supernatant. Following this, the supernatant was treated by dialysis against DI water via a dialysis membrane (100 Da) for two days to eliminate unreacted molecular materials. The yielded CQDs represented blue emission under 365-nm UV light.

### Cu^2+^ fluorescence assay and selectivity measurement

The detection of Cu^2+^ was performed in 1 mM pH 7.0 PBS by adding different cupric ion concentrations (0.001–0.1 µM for enhancement, 1–10 µM for quenching trends, respectively) to 1 ml of CQD (300 ng/ml) in PBS buffer. After a 15-min incubation, CQD fluorescence spectra were recorded at room temperature with an excitation wavelength of 300 nm. The selectivity of CQDs for Cu^2+^ ions was confirmed by adding various metal ions (e.g. CaCl_2_, Al(NO_3_)_3_, KCl, FeCl_2_, FeCl_3_, Na_2_SO_4_, CuCl_2_, HgCl_2_, PbCl_2_,AgCl_2_, ZnCl_2_, LiOH, NH_4_OH, CoCl_2_, Cr_2_O_3_, Ni(OH)_2_, MgCl_2_, and MnCl_2_) at the concentration of 10 µM) to the CQDs solution (300 ng/ml in PBS buffer pH 7.0). Then, the FL spectra were recorded after a 20 min incubation at ambient temperature.

### Analysis of a real sample

The human serum sample was prepared (from a local medical laboratory) and treated to a standard procedure^54^ to test the performance of our developed approach for the detection of Cu^2+^ in a biological environment (Table S1 Supporting Information). Initially, these samples comprising human serum were treated by the addition of CH_3_CN (1:1) and centrifuged for 15 min at 10,000 rpm to get rid of plasma proteins. Then, the obtained supernatant was diluted and added to a solution containing CQDs (300 ng mL^–1^) in PBS (pH 7.0), to which various concentrations of Cu^2+^ were added. The current research received the approval of the ethics committee at the Kermanshah University of Medical Sciences (KUMS), each participant provided informed consent. The present study was also performed in accordance with Declaration of Helsinki.

### The measurement of quantum yield

To determine the quantum yield CQDs, the integrated photoluminescence (PL) intensities and absorbance peak magnitudes of the CQD with quinine sulfate (as standard) were compared. In this line, CQDs dissolved in H_2_O, and quinine sulfate dissolved in 0.1 M H_2_SO_4_ with a known quantum yield of 54, as a standard. Initially, four various concentrations of both CQDs and quinine sulfate were prepared. In each experimental run, the absorption values tried to be under 0.1 in order to minimize the effect of re-absorption. Subsequently, FL emission spectra and UV–vis absorbance magnitudes of both CQDs and quinine sulfate samples were recorded at a consistent excitation wavelength of 300 nm. The integrated fluorescence intensity, calculated as the area under the FL curve in the wavelength range of 310–600 nm for each sample, was determined. All collected data, including absorption and fluorescence information, were utilized in the slope method to measure the relative fluorescence quantum yield of the unknown CQDs. Accordingly, the quantum yield of CQDs was measured via the below equation:

1 $$QY_{X} = QY_{ref} \left( {S_{X} /S_{ref} } \right) \, \left( {\eta_{X} /\eta_{ref} } \right).$$

In the provided equation, QY represents the quantum yield, S and *η* is the slope gained from the curves, and n stands for refractive index (*η*_*X*_/*η*_*ref*_ = 1), and the subscripts "st" and "X" refers to quinine sulfate with quantum yield of 54% and the unknown sample (CQDs).

### Computational analysis

Density functional theory dispersion corrected (DFT-D3) calculations were applied to investigate the sensing ability of the synthesized carbon quantum dot (CQD). ORCA 5.03^42^ program package was employed for doing all the quantum chemistry calculations. The structures of the CQD and its complexes with Cu^2+^, Co^2+^, and Fe^2+^ ions were optimized at the BP86-D3/Def2-SVP level of theory^55,56^. The vibrational frequency calculations were performed to calculate the thermodynamic parameters for the complexation process between CQD and different ions. It can be considered that CQD can form two types of complexes with the corresponding ions, including CQD-ion (monomer) and CQD-ion-CQD (dimer). Vibrational frequencies were employed to obtain the ground-state structures of the CQD and its complexes with different ions. These calculations revealed the nature of the stationary points as the minima with real frequencies, confirming the stability of the corresponding complexes with the metals.

The thermodynamic parameters such as Gibbs binding energy (ΔG), enthalpy (ΔH), and complexation energy (ΔE) were evaluated using Eq. (2) at 298.15 K and 1 atm.

2 $$\Delta {\text{X }}\left( {{\text{G}},{\text{ H}},{\text{ and E}}} \right) \, = {\text{ X}}_{{{\text{com}}}} {-} \, \left( {{\text{X}}_{{{\text{ion}}}} + {\text{ X}}_{{{\text{CQD}}}} } \right),$$

where the X_com_, X_ion_, and X_CQD_ are the energy parameters of the CQD complex, metal ions, and energy parameters of the free CQD, respectively.

To investigate the complexation process between the CQD and ions and the sensitivity of the synthesized CQD against the metal ions, frontier molecular orbital analysis was applied to calculate the electronic properties, including the energy of the highest occupied molecular orbital (E_HOMO_) and the energy of the lowest unoccupied molecular orbital (E_LUMO_). Using this method, it is possible to calculate quantum reactivity indices such as chemical hardness (η) and electronic chemical potential (μ). Finally, to compare and determine the nature of the interaction between the CQD and metal ions, quantum theory of atoms in molecules (QTAIM), electron localization function (ELF), and localized orbital locator (LOL) analyses were applied using MultiWFN 3.8^57–60^.

## Results and discussion

### UV–Vis and FTIR analysis

**Figure 1:**
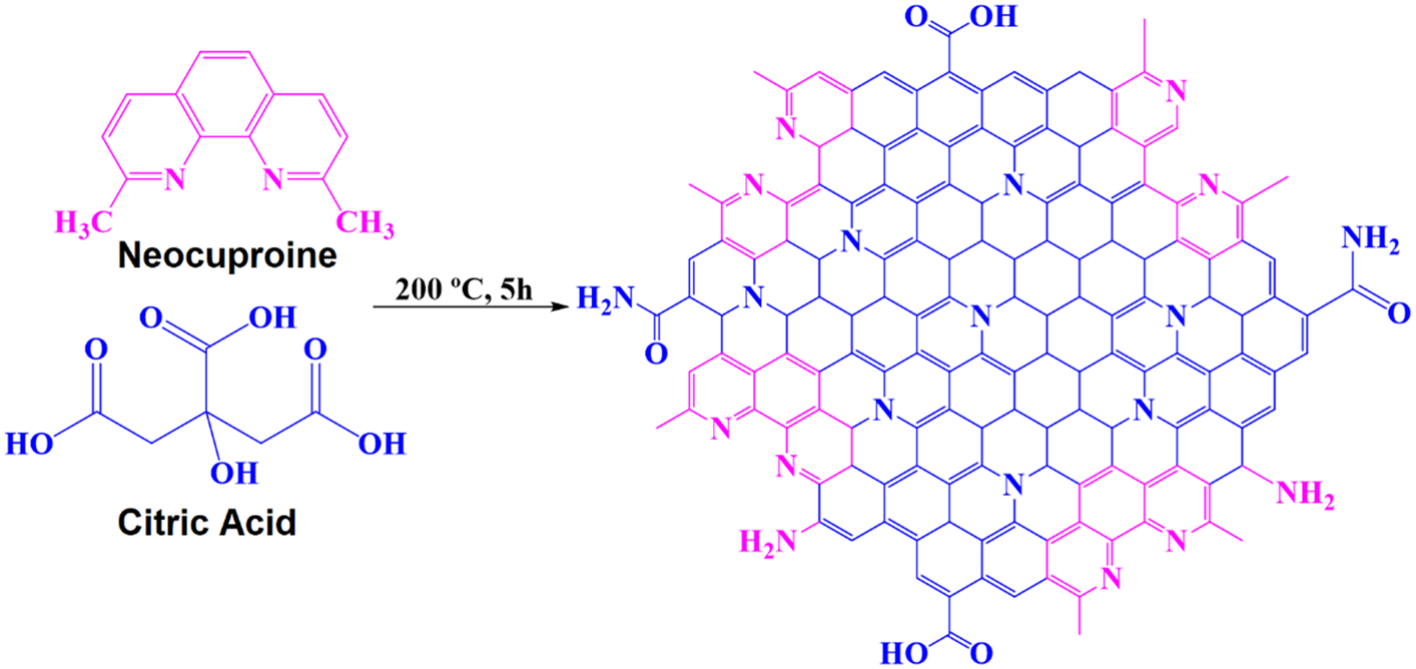
The presentation of the synthesis process of CQDs.

**Figure 2:**
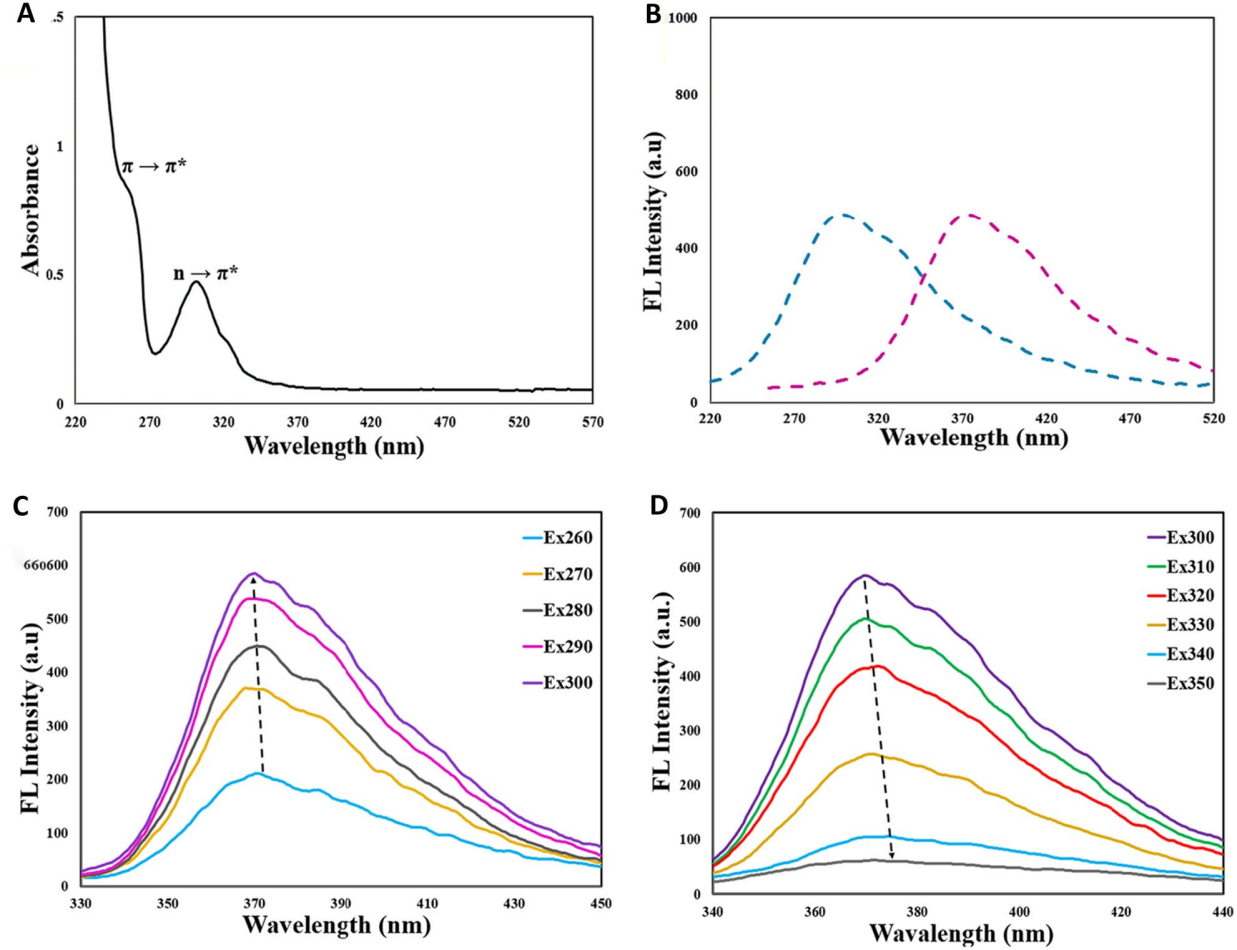
(**A**) UV–Vis absorption spectrum of the CQDs (**B**) the excitation (at 300 nm) (Blue) and emission at 370 nm (purple) of CQDs dissolved in DI water; (**C**) emission diagrams of the CQDs with increasing excitation wavelengths from 260 to 300 nm and (**D**) from 300 to 350 nm.

The successful synthesis of CQDs (Fig. 1) was approved using UV-Vis spectroscopy. The CQDs exhibited a shoulder at 240 nm, referring to the π → π* electronic transitions of the graphitic sp^2^ core, along with the wavelength of maximum absorption approximately around 275 nm stands for the n → π* transition of carbonyl bonds (or lone pair of nitrogen of pyridinic ring to carbonyl bonds). Moreover, the solution containing CQDs possessed a strong blue emission under a 365 nm UV lamp (Fig. 2A)^35^.

Photoluminescence (PL) is a widely-used, nondestructive technique for characterizing synthetic compounds. CQDs exhibited strong emission at 370 nm when excited at 300 nm (Fig. 2B). The fluorescence (FL) spectra of CQDs were examined at various excitation wavelengths from 260 to 350 nm. In this line, emission intensity increased gradually when excited between 260 and 300 nm (Fig. 2C), attributed to π to π* electronic transitions of the graphitic sp^2^ core of CQDs^61^. However, with excitation wavelengths from 300 to 350 nm, FL emission decreased (Fig. 2D), possibly due to radiative recombination of nitrogen moieties or carbonyl groups on the surface of CQDs^61^. Moreover, the highest FL emission intensity illustrated under the excitation wavelength of 300 nm (Fig. 2B).

FTIR (Fourier transform infrared) spectroscopy is a valuable tool for identifying functional groups in carbon materials. The red curve stands for FTIR diagram of citric acid (Fig. S1A supporting information), revealing strong O–H stretching and bending vibrations at approximately 3434 and 1370 cm^-1^, as well as a prominent peak at 1718 cm^–1^ related to C=O stretching in carboxylic acids. These peaks can also be observed in the FTIR spectrum of the fabricated CQDs (Fig. S1A supporting information (purple curve)). Additionally, the crimson curve is the FTIR diagram of neocuproine, indicating C–H bending vibrations and C–N stretching of the pyridinic ring at approximately 530 and 1139 cm^–1^, respectively. It is worth mentioning that these peaks can be seen in the FTIR diagram of the CQDs, which means that the pyridinic rings of neocuproine also exist in the structure of the CQDs. The purple curve in Fig. S1A supporting information also displays a strong bond at 1718 cm^–1^, associated with C=O stretching in carboxylic acids. Asymmetric and symmetric stretching vibrational modes of methyl groups stand for observed peaks from 2840 to 2930 cm^–1^, which also can be observed in the crimson curve of Fig. S1A supporting information. The skeletal stretching of C=C bonds might also result in peaks appearing at 1498 and 1509 cm^−1^. The information may cover the successful fabrication of CQDs with hydrophilic and alkylic (methyl) functional groups within their building blocks (Fig. S1A supporting information)^53,62^.

### EDS and HR-TEM analysis

**Figure 3:**
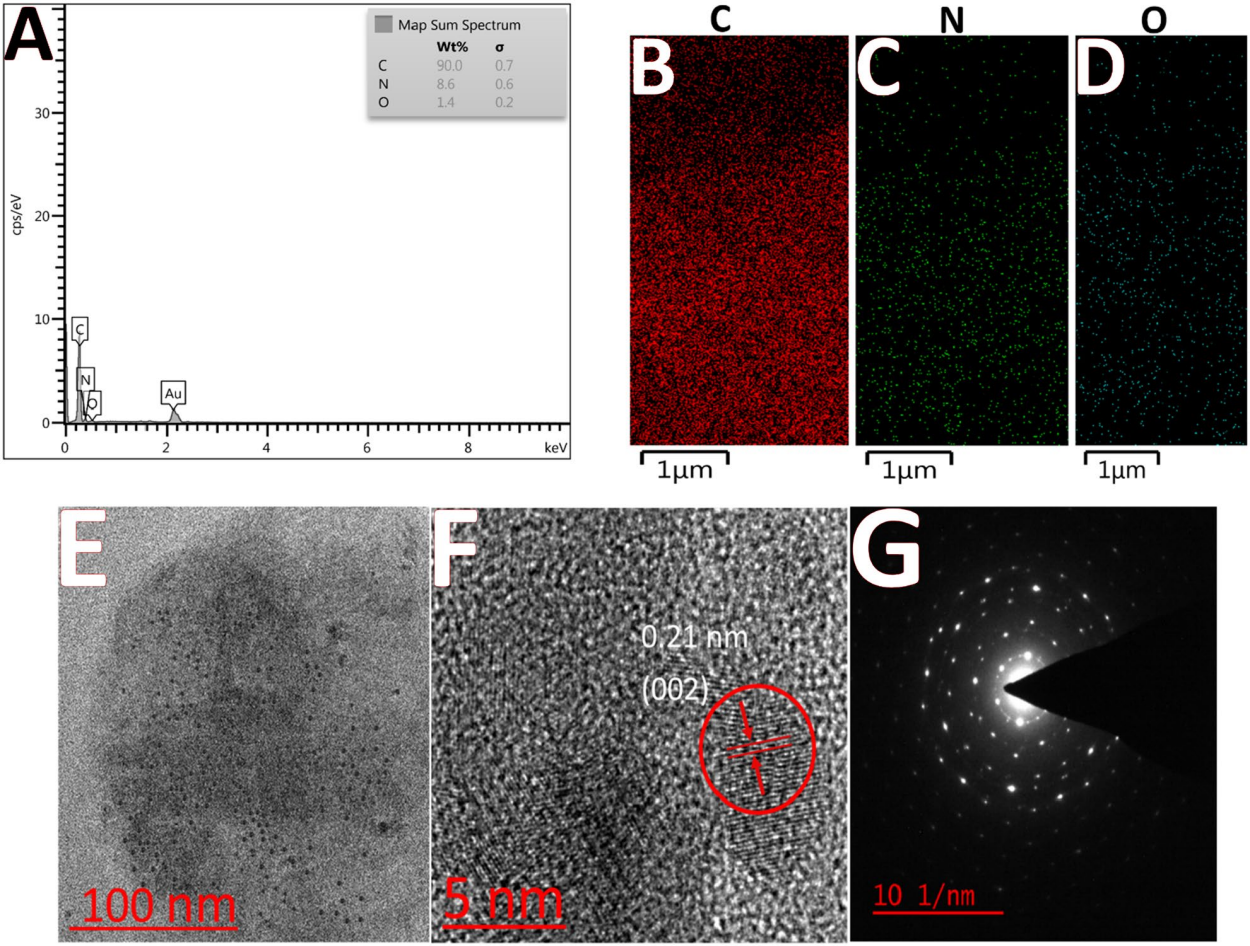
(**A**) EDS analysis of CQDs, (**B**–**D**) shows elemental mapping images of carbon, nitrogen, and oxygen, respectively; (**E**) HR-TEM image of CQDs, (**F**) 5 nm resolution of HR-TEM image of CQDs, and (**G**) SAED paradigm of CQDs.

Figure 3A displays EDS elemental mapping results of the synthesized CQDs, showing atomic percentages consistent with the reference. Additionally, the visualization highlights the spatial distribution of CQDs (Fig. 3B–D)^35^. The TEM (transmission electron microscopy) is a high significant imaging approach for characterizing nanoparticles. HRTEM (high-resolution TEM) is applied to assess CQD size distribution and morphology. In Fig. 3E, CQDs exhibit a uniform spherical shape on a smooth, well-dispersed surface, revealing a crystalline structure with clear lattice fringes. The CQD building blocks include parallel crystal planes with a typical carbon lattice and 0.21 nm uniform spacing, matching the (002) diffraction plane of graphitic (sp^2^) carbon, indicating the same core structures^35,46^ (Fig. 3F). The SAED (selective area electron diffraction) paradigm further confirms this, displaying concentric rings characteristic of graphitic (sp^2^) carbon building blocks (Fig. 3G).

### XPS and TGA analysis

**Figure 4:**
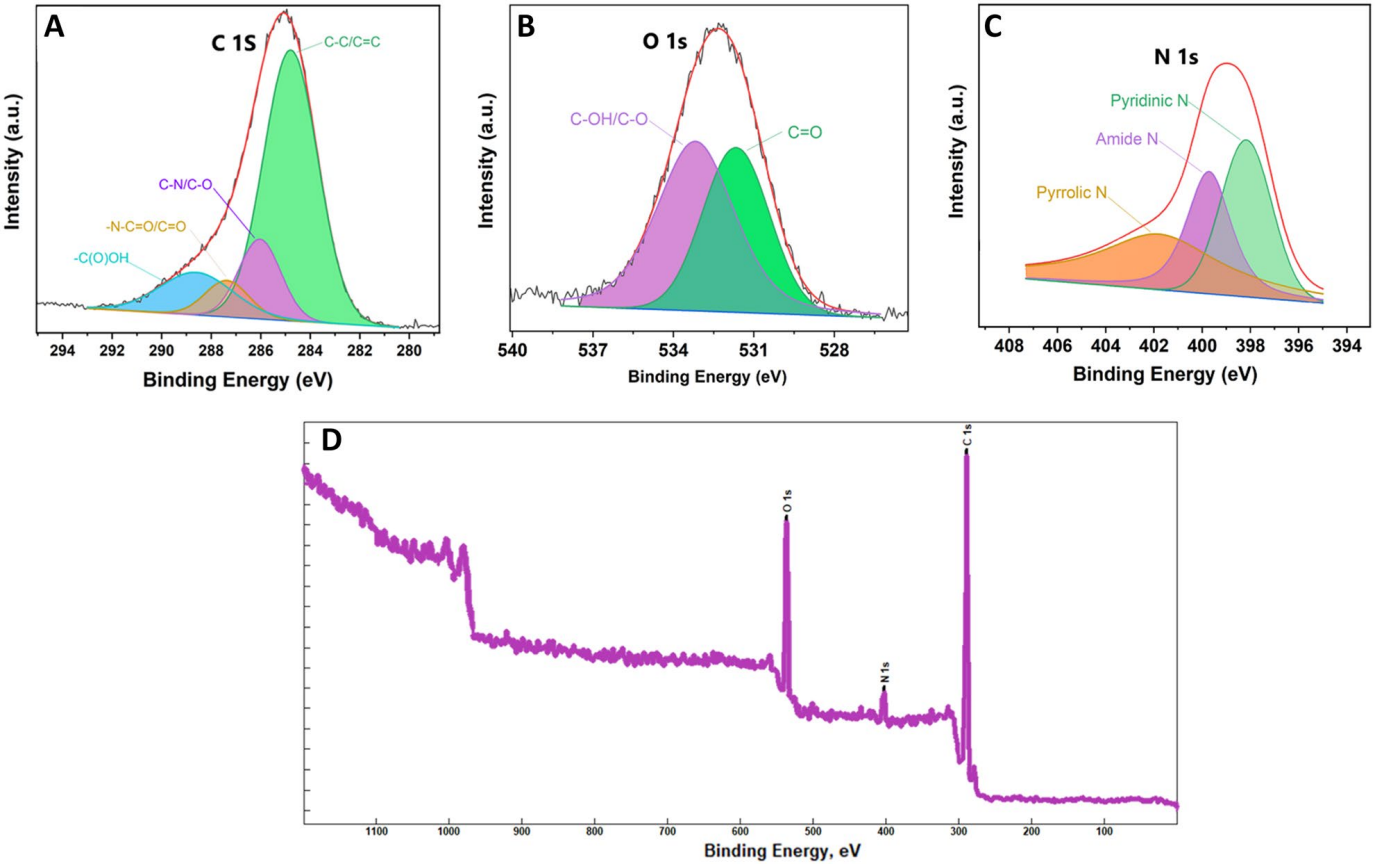
XPS analysis of as-synthesized CQDs. (**A**) High-resolution C 1 s, (**B**) high-resolution O 1 s spectra, (**C**) high-resolution N 1 s, (**D**) General XPS spectrum of the CQDs.

XPS (X-ray photoelectron spectroscopy) is a potent method for studying surface elemental composition and chemical structure (Fig. 4). Figure 4A shows the XPS spectrum of the as-prepared CQDs, comprising four main peaks in the C 1 s region: 248.30 eV (C–C/C=C), 285.9 eV (C–O/C–N), 287.45 eV (–N–C=O/C=O), and 288.4 eV (C(O)OH), correspondingly^35,53^. Moreover, XPS analysis reveals two more peaks at approximately 531.2 eV (C=O) and 533.7 eV (C–OH/C–O) in the O 1 s binding energies (Fig. 4B)^35,53,63^. The N 1 s spectrum (Fig. 4C) exhibits three main peaks at 398.2 eV (pyridinic-N), 399.9 eV (N–H of amide), and 401.9 eV (pyrrolic-N), respectively (Fig. 4C)^35,64^. Overall, the XPS data indicates that CQDs comprising carbon with a higher atomic percentage of 78.3%, oxygen (17.3%), and nitrogen (4.4%) (Fig. 4D).

To confirm the presence of various functional groups and thermal stability of CQDs, TGA (thermogravimetric) analysis at a heating rate of 10 °C min^–1^ in an inert atmosphere was supplemented (Fig. S2 supporting information). The TGA curve revealed a substantial weight loss of 90% between 30 and 720 °C. The initial weight loss at ambient temperature, approximately 5%, may be attributed to water evaporation. From 172 to 240.8 °C, there was a sharp decline, resulting in a significant weight loss of 59.1%, associated with the decomposition of hydroxyl, carboxyl groups, and covalently bonded organic components^65^. The curve showed a further weight loss of around 25% up to 405 °C, linked to the decomposition of pyridinic-like rings on the CQD surface^66^. Similar to the nature of CQDs, continuous weight loss at temperatures > 500 °C may lie in graphitic-carbon frameworks^67,68^ (Fig. S2 supporting information). In summary, these findings support the proposed CQD structure revealed by other analyses.

### ^1^H-NMR and ^13^C-NMR analysis

NMR spectroscopy is a valuable method for identifying functional groups and their adjacent substituents. In Fig. S1B supporting information, peaks in the range of 1.5–3 ppm correspond to sp^3^-hybridized alkyl protons, while those at 4–5 ppm likely represent protons linked to electronegative (O) atoms in C–OH moieties. The peaks at 6.5–8 ppm are assigned to the resonance of pyridine protons. The sp^3^-hybridized C atoms of the alkyl groups might appear at 20–40 ppm, and peaks concerned with sp^3^ C atoms bound to electron withdrawing (O) atoms are seen at 70 ppm. The Chemical shifts for aromatic carbons and pyridine rings can be identified within peaks ranging from 120 to 152.9 ppm. Additionally, peaks between 172.6 and 175.6 ppm confirm the presence of carbonyl groups (Fig. S1C supporting information). These data and analyses support the successful preparation of CQDs using the synthetic method.

### Setup the fluorescence (FL) technique for the detection of Cu^2+^

Trapped excited states in CQDs produce potent fluorescence at 370 nm upon 300 nm excitation. CQDs exhibit strong emissions in the range of 260 to 360 nm excitation, with a peak at 370 nm when excited at 260 nm and even stronger emissions when excited at 270–290 nm, peaking at 370 nm. The most intense fluorescence is observed at 300 nm excitation (Fig. 2D). Emissions decrease significantly from 310 to 360 nm excitation, maintaining the same peak position but reaching negligible intensity at 360 nm (Fig. 2D). CQD fluorescence likely arises from electronic transitions at the boundary between oxidized and non-oxidized carbon regions, influenced by size and surface states^69–72^. XPS analysis confirms the presence of N, C, and O on the CQD surface (Fig. 4), indicating diverse surface states with varying energy levels and emissive traps. Remarkably, the water-soluble CQDs maintain strong and stable fluorescence at ambient temperature, remaining unchanged after a year of storage.

As noticed before, the changes in CQDs' fluorescence via Cu^2+^ ions may lie in the formation of complexes between Cu^2+^ ions and CQDs' surface heteroatoms, causing changes in CQDs' excitation and emission properties. Accordingly, there is a relationship between the concentration of Cu^2+^ and the CQDs’ FL to establish a quantitative analysis of Cu^2+^. Initially, the optimum concentration of CQDs for Cu^2+^ sensing is adapted before considering the Cu^2+^ concentration effect on the FL of CQDs. The FL intensity of CQDs exhibited an excellent linear response from 0 to 300 ng mL^–1^, whereas it deviated from linearity at higher concentrations exceeding 300 ng mL^–1^ (Fig. S3 supporting information). Hence, the CQDs' concentration for Cu^2+^ determination was set at 300 ng mL^–1^. Additionally, the optimum Cu^2+^ concentration range in the presence of CQDs (300 ng mL^–1^) is established. In this line, the CQDs' fluorescence response remained stable when cupric ions were added at concentrations exceeding 0.1 µM for "Turn-on" and 10 µM for "Turn-off" states (Fig. S4A–D supporting information). The most linear responses were observed within the cupric ion addition ranges of 0.001–0.1 µM and 1–10 µM for both observed phenomena, respectively. On the one hand, in the vicinity of a quencher with a specific concentration, a lower concentration of the fluorophore generally gives rise to a higher change (F/F_0_), and eventually, higher sensitivity can be achieved^73,74^. On the other hand, S/N (signal-to-noise) ratio may be decayed as far as too much low fluorophore concentration is used. Eventually, 300 ng mL^–1^ CQDs were adapted for Cu^2+^ detection with a wide linear range and the lowest limit of detection. Moreover, the CQDs FL intensity is meaningfully turned-off as much as 45.4%, and however, turned on by 64.7% after 10 µM, and 0.1 µM Cu^2+^ addition to CQDs (300 ng mL^–1^) solution, remaining stable for approximately one h experiment (Fig. S5 supporting information). These observations signify that the quenching/boosting of the FL of CQDs via Cu^2+^ is fast and stable, suggesting a promising sensor for high-sensitive- and -selective detection of Cu^2+^.

**Figure 5:**
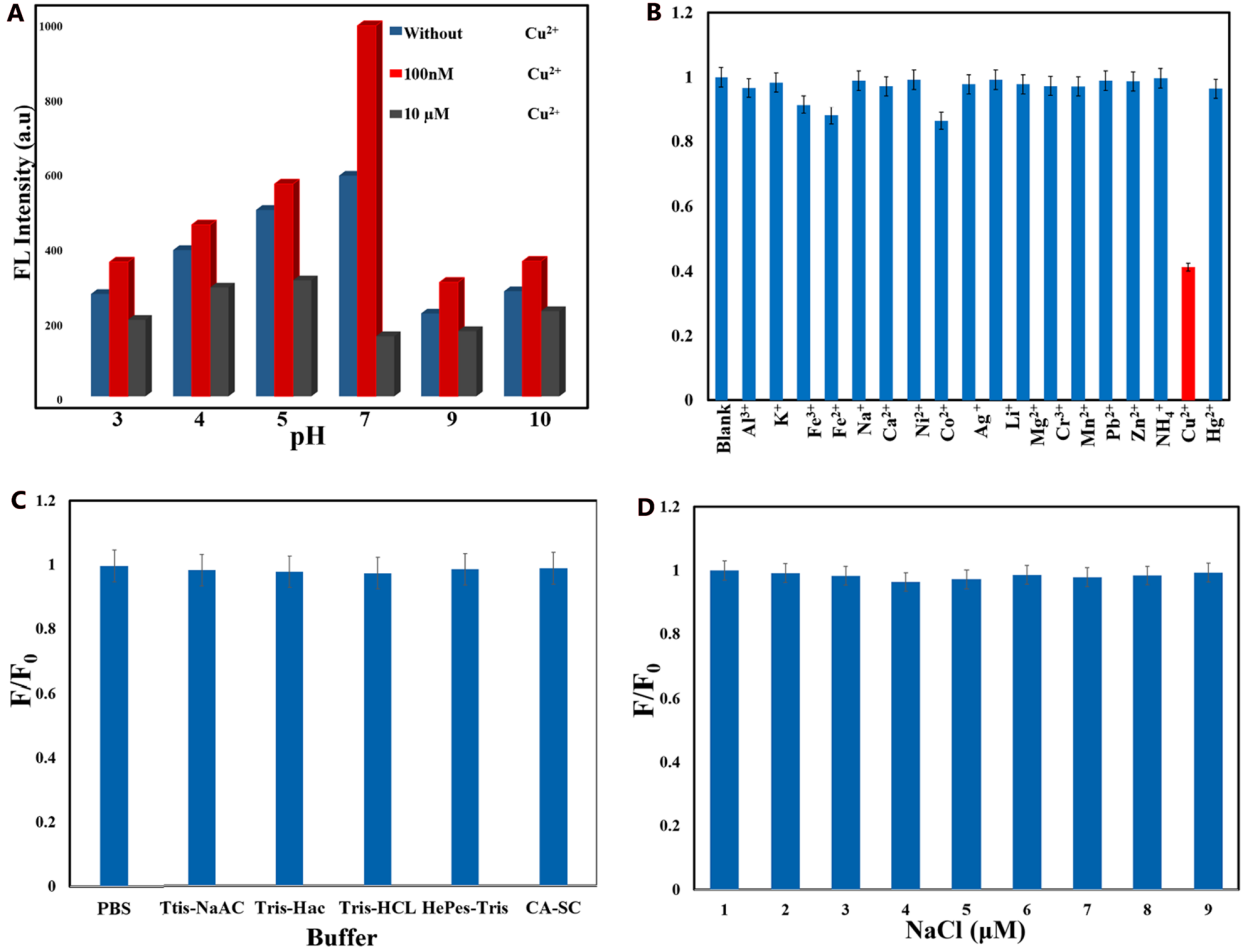
(**A**) FL responses of CQDs (300 ng mL^-1^) in the absence (blue) and presence of 0.1 μM (red) and 10 µM (gray) cupric ions at various pH values. (**B**) Selectivity of the CQD-based probe for copper ions over other ions (Al^3+^, K^+^, Fe^3+^, Fe^2+^, Na^+^, Ca^2+^, Ni^2+^, Co^2+^, Ag^+^, Li^+^, Mg^2+^, Cr^3+^, Mn^2+^, Pb^2+^, Zn^2+^, NH_4_^+^, Cu^2+^, and Hg^2+^) in pH 7.0 PBS solution (the concentrations of CQDs and metal ions were 300 ng mL^-1^ and 10 μM, correspondingly). The photostability of CQDs (300 ng mL^-1^ in various conditions; (**C**) buffer effects; (**D**) NaCl with various concentrations (1–9 µM).

Another crucial factor for Cu^2+^ detection using our sensor is the solution's pH, as it affects the fluorescence of CQDs in the presence of cupric ions. CQDs exhibited strong fluorescence in the pH range of 5–7, especially at pH 7, while fluorescence was weak at alkaline conditions (pH 9, 10). This suggests that a slightly acidic environment (pH 5–7) is ideal for sensing^73^. Surprisingly, two interesting phenomena happened in the acidic and alkaline pH after the addition of CQDs; the addition of 0.1 µM cupric ions led to FL enhancement (turn-on), although 1 µM addition of this ion made CQDs be quenched (turn-off). While these two phenomena were observed in a wide pH range, the quenching/boosting coeficiencies (F/ F_0_) at various pH values quite varied. In high acidic pH (pH ≤ 3), cupric ions additions (10 µM) have approximately no impact on the CQDs’ FL, which might be associated with the well-protonation of existing heteroatoms (N-pyiridinic ring, carboxyl, and hydroxyl groups) at the CQDs’ surface and finally they cannot get engaged in complexation with cupric ions to prepare the FL-quenching Cu^2+^ ions-heteroatom complexes^73^. In the pH above 7.0, the quenching coeficiencies are less satisfactory, likely because of the potential incomplete hydrolysis of cupric ions, which inhibits the formation of complexes between Cu^2+^ ions and existing heteroatoms of CQDs’ surface^73^. The quenching coefficiencies are notably high in the slightly acidic pH range (5–7), suggesting that the optimum pH for the sensitive Cu^2+^ determination lies within this range. However, considering the highest fluorescence intensity and the most significant quenching/enhancement coeficiencies (F/F_0_), pH 7 appears to be the best choice for our sensing job (Fig. 5A). The selectivity of the CQDs’ FL sensing was investigated in which the effects of metallic cations such as Ca^2+^, Al^3+^, K^+^, Fe^2+^, Fe^3+^, Na^+^, Cu^2+^, Hg^2+^, Pb^2+^, Zn^2+^, Co^2+^, Ni^2+^, Li^+^, Mg^2+^, Mn^2+^, NH_4_^+^, and Cd^2+^ on the CQDs’ FL were tested. In Fig. 5B, when 10 µM of various metal ions were added to the CQDs solution, most of them had no notable impact on CQDs' fluorescence, except for Cu^2+^, which caused a significant decrease in fluorescence compared to the other metal ions. This indicates that our probe exhibits strong selectivity for Cu^2+^ over other metal ions. However, Fe^2+^ showed a slight ability to quench CQDs' fluorescence under the same aqueous medium (pH 7). Nonetheless, the sensing performance of our probe experiences a slight decline in weakly acidic conditions (pH 5). For instance, besides Fe^2+^ exhibiting moderate quenching, both Fe^3+^ and Co^2+^ can interfere with Cu^2+^ detection at pH 5 (Fig. S6 Supporting Information). At alkaline pH (8–10), the heteroatoms on the CQDs' surface might become deprotonated, while they get protonated at strongly acidic levels (pH 3, 4). This disrupts the complexation of metallic ions, particularly Cu^2+^, with the CQDs' surface heteroatoms, causing a decrease in our CQDs' selectivity and performance in harsh acidic and basic environments.

The quantum yield is a measure of the ratio of emitted photons to absorbed photons in a sample. The comparative method, as expressed in Eq. (1), is a well-established and reliable approach for determining fluorescence quantum yield. This method involves comparing the wavelength-integrated intensity of the test sample with that of a standard sample. To enhance measurement accuracy, the absorption range of the standard sample should align with the excitation wavelength range of the test sample. The quantum yield is calculated by analyzing the variation of integrated fluorescence spectrum intensity with absorbance at the excitation wavelength of both the test and standard samples, utilizing Eq. (1). Interestingly, the quantum yield of the CQDs was calculated to be 33.65%, showing that the use of citric acid and neocuproine as precursors could enhance the CQDs quantum yield. Accordingly, pH 7.0 is selected as an optimum pH for the Cu^2+^ determination of in a real water sample to minimize potential interferences. Following the optimization of CQDs concentration, pH, and selectivity of the sensor, we will explore the impact of salt concentration and different buffer effects on the stability of CQDs' fluorescence. Different buffers (such as PBS, Tris-HAc, Tris-NaAc, Tris–HCl, Hepes-Tris, and CA-SC) were applied to check out the photostability of CQDs (300 ng mL^-1^), showing no clear inference with FL of CQDs (Fig. 5C). Moreover, approximately no meaningful changes in the CQDs’ FL intensity were observed at various concentrations of NaCl (1–9 µM, pH 7) (Fig. 5D). These two experiments highlight the excellent photostability of our CQDs in dealing with inorganic salt interference (Fig. 5D). Sensitivity, detection limit, and linear range response of the CQDs-based determination framework were then investigated through optimized experiments.

**Figure 6:**
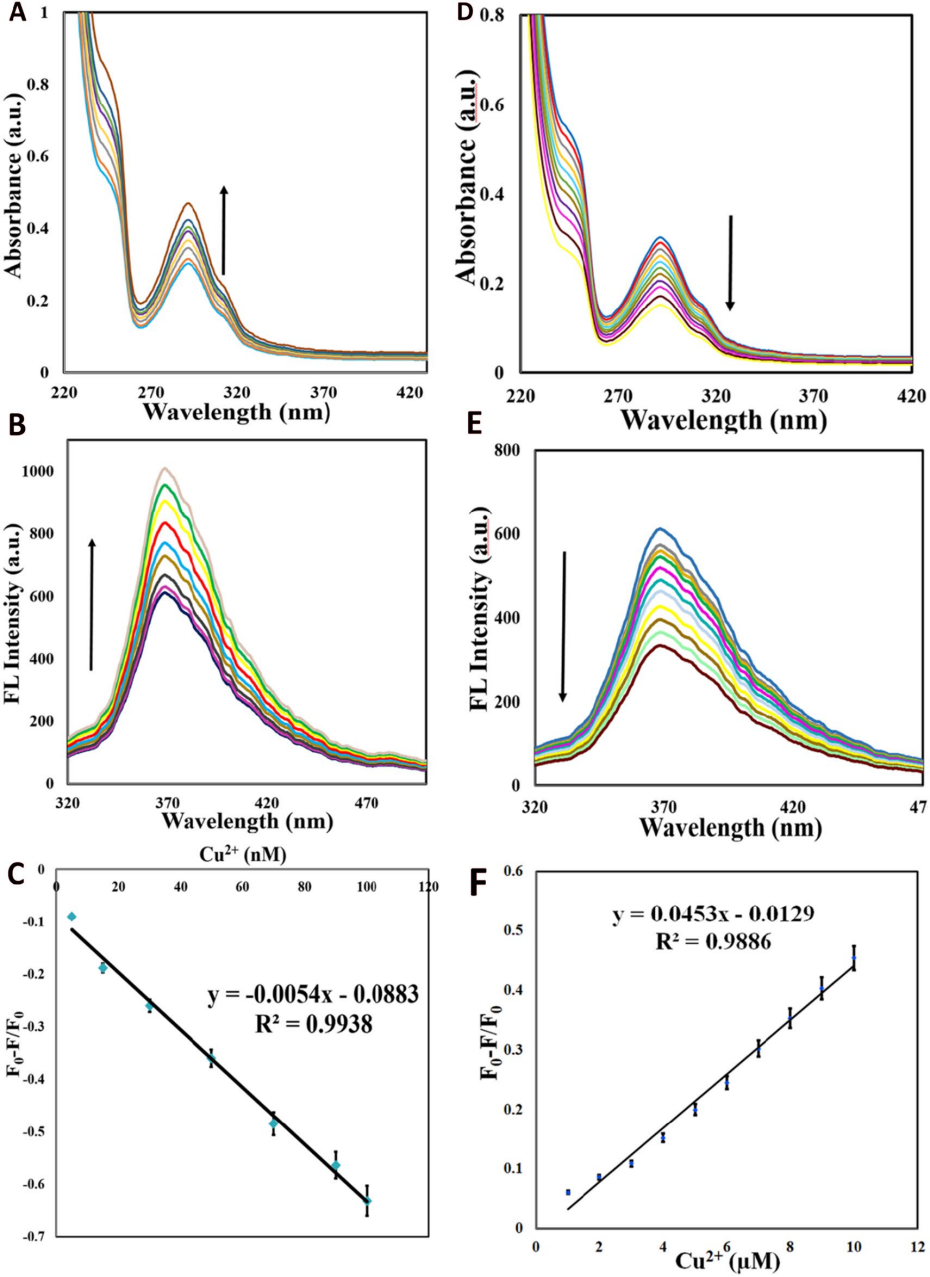
UV, FL response of CQDs (300 ng mL^-1^), and the plots of F_0_-F/F_0_ of CQDs (300 ng mL^-1^) vs. the Cu^2+^ concentration under the addition of different concentrations of Cu^2+^ ions (from top to bottom); (**A**–**C**) 0.001, 0.005, 0.15, 0.03, 0.05, 0.07, 0.09, 0.1 µM (**D**–**F**) 1, 2, 3, 4, 5, 6, 7, 8, 9, 10 μM in a pH 7.0 PBS solution.

As exhibited in Fig. 6, CQDs' fluorescence is significantly influenced by Cu^2+^ concentrations, leading to two intriguing phenomena at two various concentrations of cupric ions. Initially, the nano-molar range addition of Cu^2+^ ions (from 0.001 to 0.1 µM) left the FL intensity of CQDs to experience a linear increase, whereas micro-molar addition of this ion (1–10 µM) led to fully linear-quenching of CQDs’ FL. A strong linear relationship exists between Cu^2+^ concentration and boosting/quenching efficiencies (F_0_-F/F_0_) within the ranges of 0.001–0.1 µM (turn-on) and 1–10 µM (turn-off). The F and F_0_ stand for the FL intensity of CQDs in the presence and absence of cupric ions. Interestingly, the same phenomena were screened by UV–Vis analysis through the same conditions (Fig. 6).

It is important to note that the probe showed a detection limit of 0.001 µM for Cu^2+^. These results suggest that the as-prepared CQDs with potent FL properties may be a promising probe for Cu^2+^ sensing. Moreover, we have compared the detection limit and linear range of our CQD-based sensor with those reported in the literature (Table 1). Interestingly, the findings of this study revealed that although our probe might have a narrow pH range somehow, our CQD-based sensor exhibited a much lower LOD in comparison to the other previously reported methods. Moreover, our designed sensor has also shown a comparable wider linear range. Furthermore, our probe is the only repoted sensor by which Cu^2+^ ions can be detected in two different ranges through two distinctive phenomena, making this sensing framework an appealing platform for Cu(II) sensing in real water and in human serum samples, and can be a viable tool for Cu^2+^ detection in the serum of individuals suffering from WD.

**Table 1:** Various sensors for the Cu^2+^ ions determination.

Sensing device	LOD (μM)	Linear range (μM)	Ref
CQDs	0.008	0–0.225	^75^
CQDs	1.5	–	^76^
Sulfur QDs-CQDs	0.031	0.1–5.0	^77^
CQDs/Au NCs	0.013	0.025–4.0	^78^
CdTe QDs	0.055	0–20.0	^79^
CQD/Si NPs	0.035	0–3.0	^80^
CQD/Si NPs	0.01	0–1.0	^81^
CQDs	0.007	0.01–1.0	^82^
Cys-CdS QDs	0.34	0.50–2.25	^83^
NAC-Mn:CdS QDs^Δ^	0.041	0.16–3.36	^84^
CQDs	0.001	0–0.1 (Turn-on), 1.0–10.0 (Turn-off)	This work

^Δ^NAC-Mn:CdS QDs, N-acetyl-L-cysteine capped Mn:doped CdS quantum dots.

### Copper sensing in the plasma samples of healthy and affected individuals by Wilson’s disease

**Figure 7:**
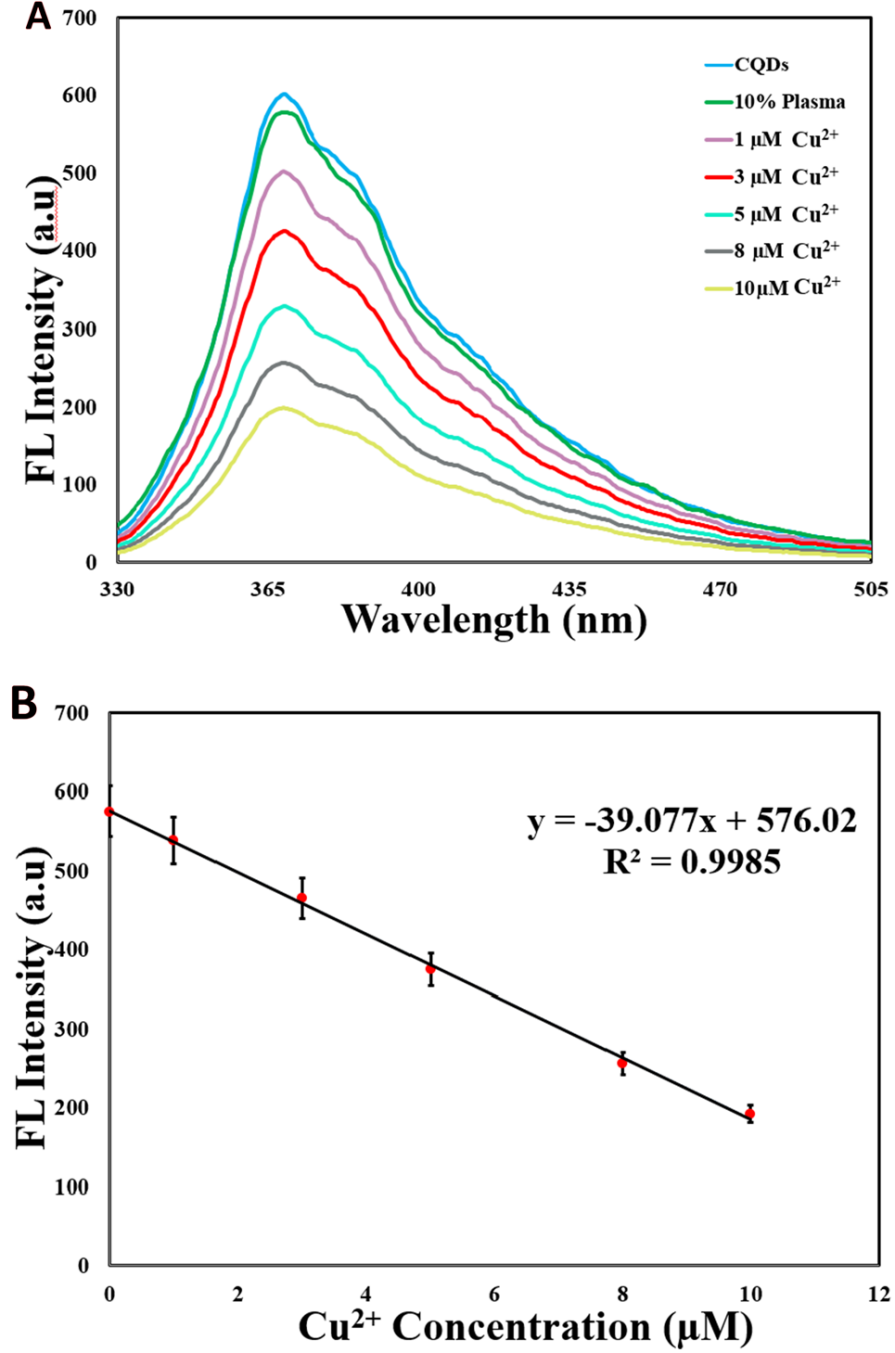
Sensing of Cu^2+^ in the plasma of individual affected by WD using the CQD-based system. (**A**) FL spectra (λ_ex_ = 300 nm) of CQDs (300 ng mL^-1^ μM) upon addition of cupric ions (1–10 µM) to 10% deproteinized plasma of individual affected by WD diluted with pH 7.0 PBS buffer; (**B**) Calibration curve of CQD-based sensor (300 ng mL^-1^) µM at pH 7.0 PBS (PBS buffer, 50 mM) revealing a linear correlation between FL intensity (λ_ex_ = 300 nm, λ_em_ = 375 nm) and Cu^2+^ concentration (1–10 µM). (The emission spectra and calibration curve were taken 4 min after mixing).

To assess the reliability and applicability of the CQD-based sensor, we also performed a test to determine the amount of copper in human serum samples healthy and affected individuals by Wilson’s disease. The 10% deproteinized human serum samples were treated based on the mentioned procedure in the experimental section. According to our results for the detection of copper in the serum sample of healthy individual, interestingly, a linear relationship (y = − 29.614x + 620.57, R^2^ = 0.9986) between CQDs’ FL quenching and copper concentration was observed, proving that our probe works well even in the presence of human plasma. The changes in the CQDs’ fluorescence (replicate n = 5) and Cu^2+^ spiked samples were determined underlying the procedure mentioned above, and the copper present in the plasma was obtained through the regression equation of the standard calibration curve (Table S2 supporting information, Fig. S7 Supporting information). Hence, the content of the copper in the serum of healthy volunteer (after 2–10 µM cupric ions addition) was found to be 22.451 µM, which is astonishingly well fit in the range of cupric concentration in the plasma of healthy individuals^85,86^. Recoveries of the known spiked amounts of cupric ions were between 99.8 and 101.5%, showing a good accuracy of the sensing platform. Astonishingly, the RSD% magnitudes for all measurmentes were seen to be lower than 6%, suggesting that our sensor possesses a convincing precision. These results inspired us to quantitatively determine copper in the serum of a volunteer affected by WD using the same protocol. Underlying our findings, a good linear relationship (y = − 39.077x + 576.02, R^2^ = 0.9985) between CQDs’ FL quenching and Cu^2+^ concentration was also observed (Fig. 7). The concentration of copper in the plasma was then determined using a regression equation obtained from a standard calibration curve. Moreover the measeured concentration of Cu^2+^ by our CQD-based probe was also compared by those obtained by ICP-OES technique (Table 2). The measured concentration of cupric ions (after the addition of 1–10 µM) to the plasma samples was determined to be 8.1236 µM (Table 2). It is noteworthy that this value aligns remarkably with the observed range of cupric concentration in the plasma of affected individuals by WD^14,87,88^. Recoveries of the spiked amounts of Cu^2+^ ions varied from 94.7% to 102.9%, and also, the RSD% values for all measurmentes were seen to be lower than 6%. These results indicate that the CQD-based sensor possesses a good accuracy and a convincing precision. Significantly, our CQD-based probe demonstrates a strong correlation with ICP-OES in measuring Cu^2+^ levels in WD-affected serum, underscoring its potential for precise Cu^2+^ quantification. In total, the selective, and sensitive CQD-based probe is a reliable and precise option for monitoring copper concentration, making it a valuable tool for diagnosing Wilson's disease in clinical settings.

**Table 2:** Determination of Cu^2+^ content in 10% deproteinized human plasma samples. (Linear range of µM addition of cupric ions-quenching trend).

Spiked (µM)	Proposed technique			ICP-OES		
	Measured (µM)	Recovery (%)	RSD (%)	Measured (µM)	Recovery (%)	RSD (%)
0	0.0414 ± 0.00107	–	2.6	0.0420 ± 0.00075	–	1.8
1	0.972 ± 0.03697	97.2	3.8	0.991 ± 0.02675	99.1	2.7
3	2.841 ± 0.11649	94.7	4.1	2.925 ± 0.08482	97.5	2.9
5	5.144 ± 0.19033	102.9	3.7	5.118 ± 0.09212	102.4	1.8

### Paper-based Cu^2+^ determination

**Figure 8:**
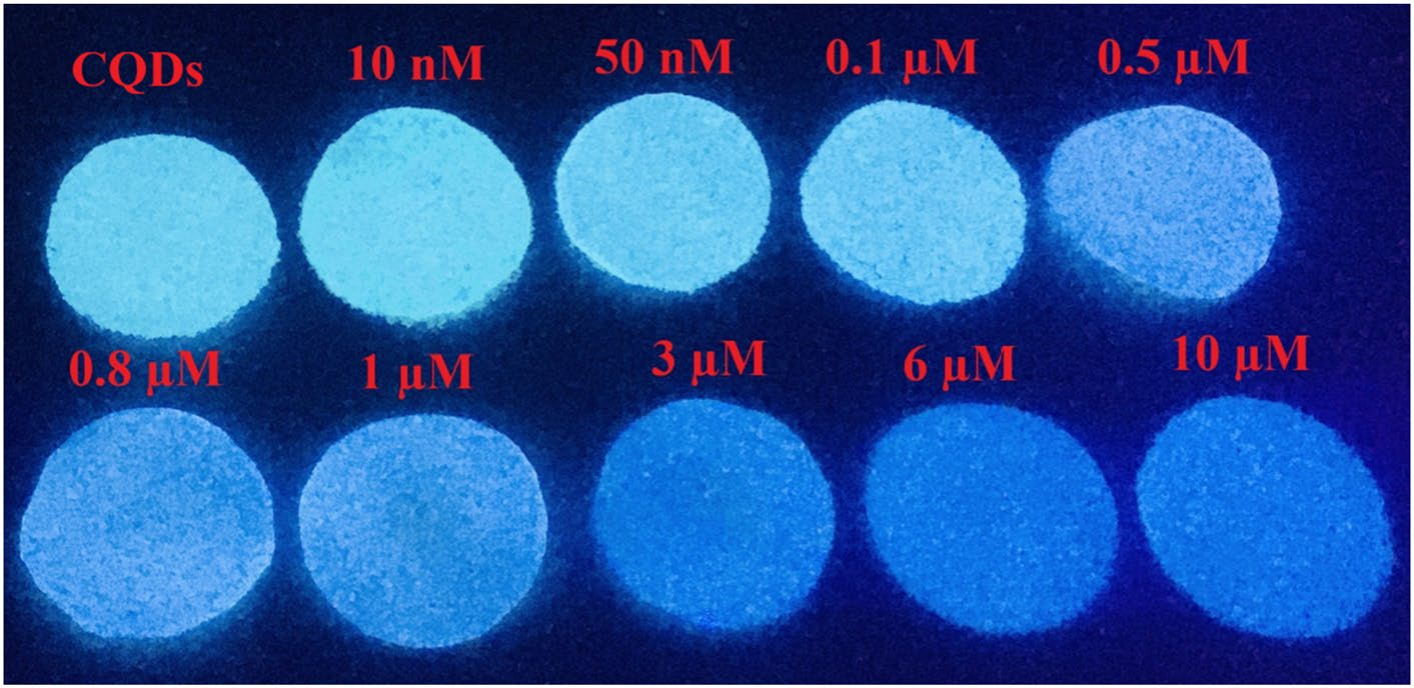
The photographs of the paper-based test under a 365 nm UV lamp. The concentrations of Cu^2+^ from left to right: 0, 0.01, 0.05, 0.1, 0.5, 0.8, 1.0, 3.0, 6.0, 10.0 µM.

Our probe is capable of Cu^2+^ determination not only in aqueous solution, but also could be applied for a significant color difference in the paper. Initially, a bunch of uniform filter papers was prepared approximately 10.05 mm in diameter for paper-based detection of Cu^2+^. For a practical paper-based test, a sufficient volume of the standard solution (100 µL) comprising CQDs was syringed on the as-prepared filter papers. After that, they were kept to be naturally dried at ambient temperature. Afterward, the color variations resulting from Cu^2+^ addition were recorded under a 365 nm UV lamp. As illustrated in Fig. 8, dropping Cu^2+^ solution onto the papers showed interesting FL-color changes, which relies on the amount of added Cu^2+^ solution onto the paper; the nano-molar addition range (< 0.1 µM) causing an observable brightness while the micro-molar addition range led the color to become more darker, in general. The addition of 0.01–0.1 µM caused the color to become brighter before getting a little darker with the addition of 0.5 µM. After adding cupric ions onto the paper as much as 0.8–1 µM, the color did not experience an intense change, after which the color got darker from 3 µM on, and became navy blue after the addition of 10 µM Cu^2+^ ions (Fig. 8). According to paper-based test results, it is clear that the high sensitivity of our CQD-based probe, even on paper, makes a promising, fast, and on-site sensor for Cu^2+^ determination without any sophisticated instruments.

### Mechanism of FL emission quenching and enhancement of CQDs

**Figure 9:**
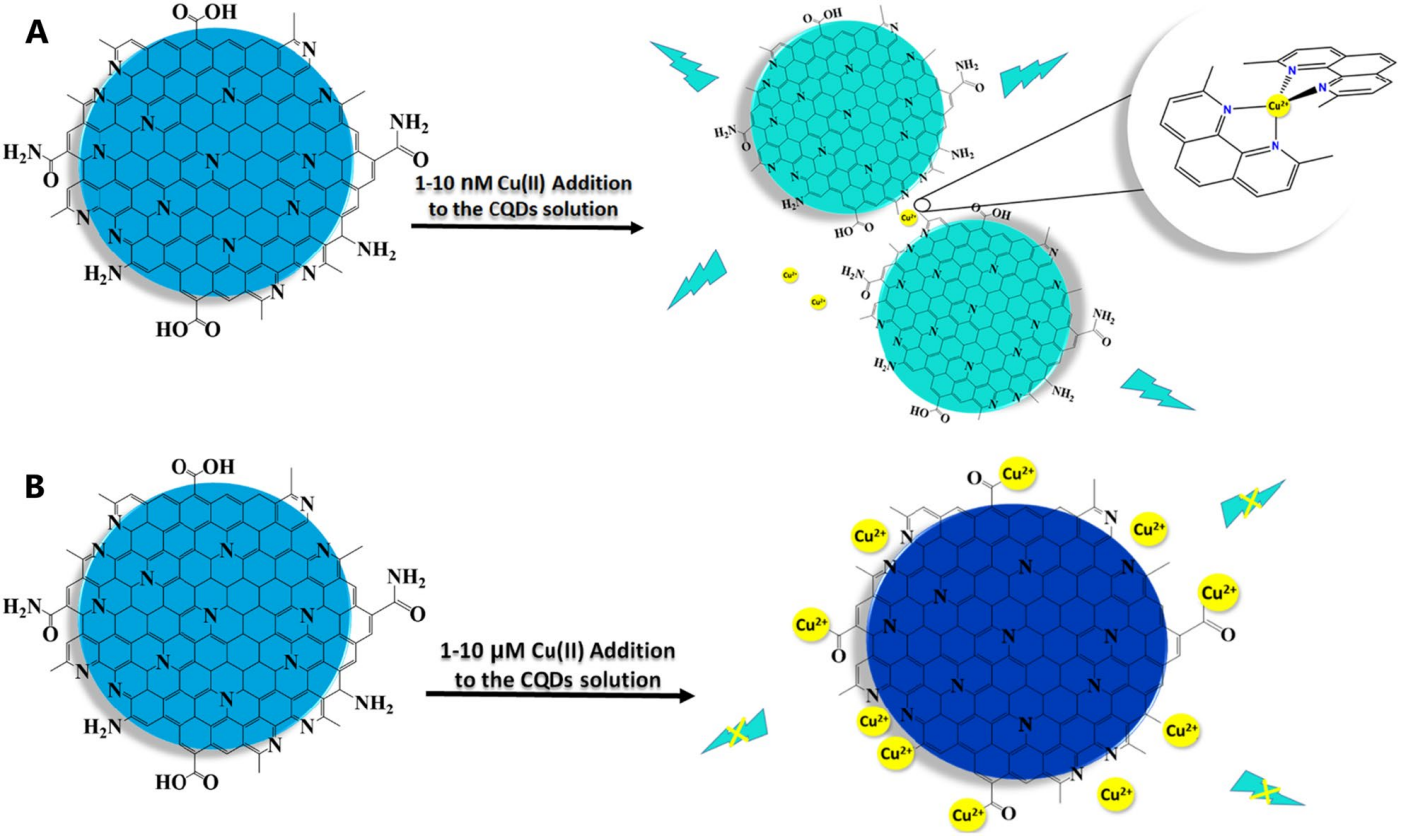
The mechanism of (**A**) Turn-on state under the addition of 0.001–0.1 µM Cu(II) and (**B**) and Turn-off state under 1–10 µM Cu(II).

The prepared CQDs (from Neocuproine and citric acid as precursors) are well-dispersed with spherical morphology (Fig. 3), and a size of < 10 nm, exhibiting potent blue emission under the excitation of 300 nm. The strong blue emission of CQDs has resulted from Cu^2+^ addition (in the range from 0.001 to 0.1 µM), the so-called ‘’Turn-on’’ state, whereas it experienced a decreasing trend (‘’Turn-off’’ state) after the addition of 1–10 µM Cu^2+^. However, both ranges of cupric ions additions would not affect the FL wavelength. Moreover, UV–Vis spectra of CQDs delineate two absorption peaks at around 240 and 300 nm, which experienced a rise in intensity after adding Cu^2+^ (in the range of 0.001–0.1 µM), however, it dropped when 1–10 µM Cu^2+^ ions were added (precisely as it was observed in FL data) with no changes in wavelength (Fig. 6). There is an excellent linear correlation between the Cu^2+^ concentrations and quenching/enhancement co-efficiencies. There might be logical explanations for these interesting observations. It can be speculated that the existence of heteroatoms (i.e. N, O) as chelate groups on the CQDs surface pose a strong affinity to make a complex with Cu^2+^, which in turn can make these CQDs a high-selective and sensitive probe for the identification of cupric ions^73,89,90^. As for the mechanism of both the turn-on and turn-off states (or FL enhancement and quenching), after the addition of cupric ions in the range of 0.001–0.1 µM and 1–10 µM, the complexation states of Cu^2+^ with CQDs surface agents might have significant roles. It is important to mention that the mechanism of quenching (turn-off state) may tie in e^-^-transfer or energy transfer^46,73^, meaning that cupric ions can play a role as e^**-**^-acceptor groups and CQDs having lots of various functional groups such as π-systems, carbonyl and hydroxyl moieties, N in aromatic rings and amide groups on their surface act as e^-^-donor groups, from one hand. On the other hand, Cu^2+^ ions are paramagnetic ions possessing unoccupied d orbitals by which the adsorption of Cu^2+^ on the surface of CQDs can be facilitated through the complexation of heteroatom-containing functional groups at the surface of CQDs and d orbitals of Cu^2+^ ions, finally, the turn-off phenomenon is observed^46,91–93^ (Fig. 9B).

However, another story might be cause of the FL-enhancement (Turn-on state) phenomenon. As mentioned before, our CQDs were fabricated via neocuproine and citric acid. As is apparent, the neocuproine bearing pyridinic rings with high potential for complexation with cupric ions^94,95^. Hence, the pyridine-like rings of this ligand might be possibly located at the surface of as-prepared CQDs (Fig. 9), making the behavior of CQDs similar to neocuproine toward Cu^2+^ ions. As far as cupric ions are added in a low concentration range (from 0.001 to 0.1 µM), these ions make complexes with the nitrogens lone pairs of pyridine-like rings located at the surface of CQDs due to the tendency of Cu^2+^ ions to surface-located pyridine-like rings. These complexations may give rise to keep closer two or more nanoparticles to each other (or dimerization may occur), leading to rigidity in their structures. The rigidity in the building blocks of CQDs may possibly raise the electron and charge transfer. Finally, brighter emission will possibly be created (Fig. 9). This might be a likely explanation for FL-boosting of CQDs by cupric ions addition in the nano-molar range (0.001–0.1 µM). Moreover, in this range, the other hetero-atoms at the surface of CQDs might be free (because of the high tendency of Cu^2+^ to pyridinic rings), making the possibility for their electrons to be excited, eventually, increasing the FL emission of CQDs (Fig. 9A). This term will be changed as the concentration of Cu^2+^ ions increases in the solution, and cupric ions make complex with all heteroatoms at the CQDs’ surface and make their FL emission to be quenched.

**Figure 10:**
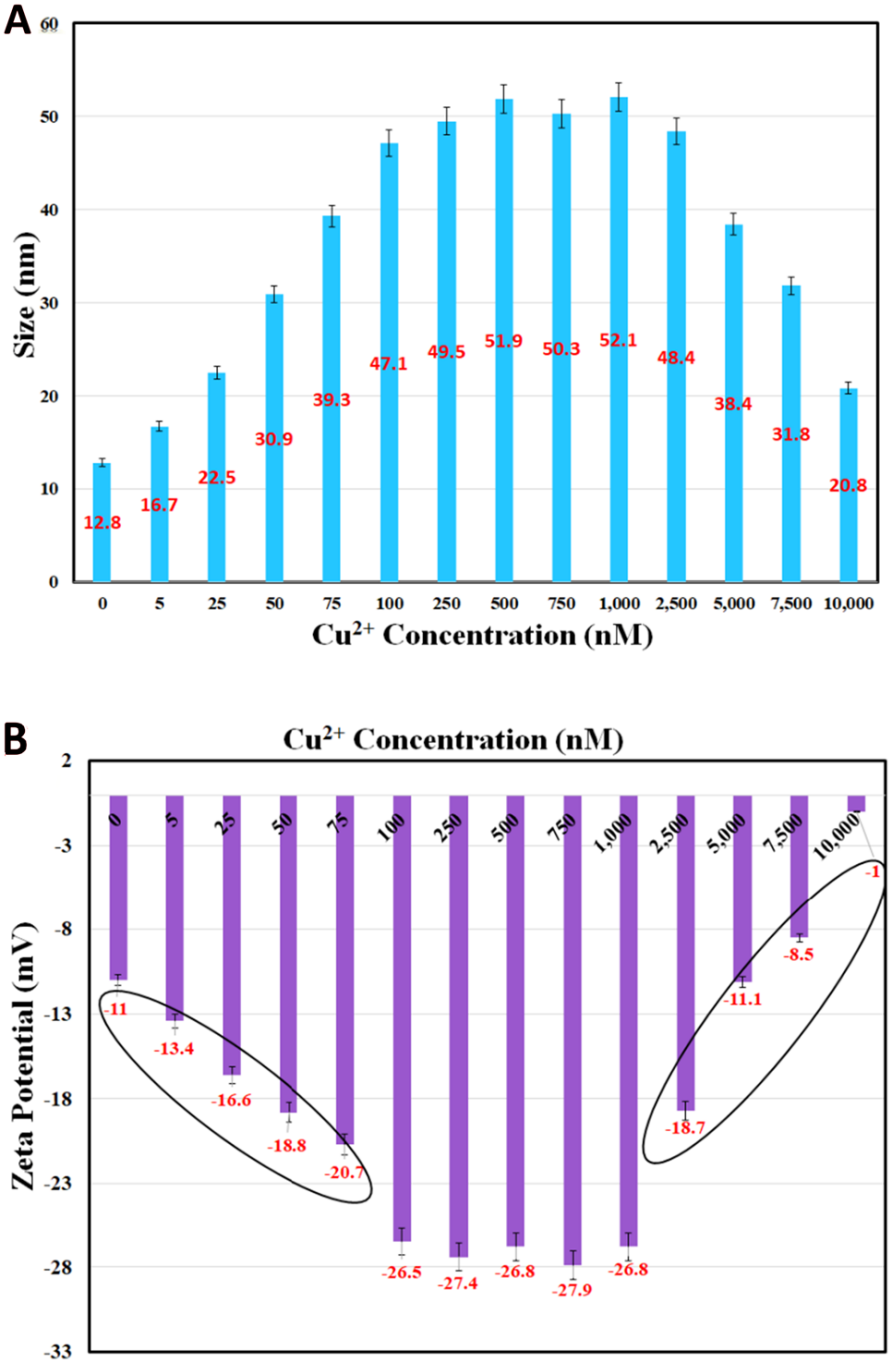
(**A**) DLS and (**B**) Zeta potential of CQDs in the absence and presence of 0.005, 0.025, 0.050, 0.1, 0.25, 0.05, 0.75, 1.0, 2.5, 5.0, 7.5, and 10 µM of Cu^2+^.

In order to back up our suggested mechanism for both observed phenomena, the DLS and ζ-potential analysis of CQDs in the presence and absence of cupric ions were taken (Fig. 10). According to the DLS analysis, the size of CQDs stands for 12.8 nm, which this number experienced significant changes after different concentrations of cupric ions are added to the solution containing CQDs. The size of CQDs showed a remarkable increase after the addition of 0.005, 0.025, 0.050, 0.075 µM cupric ions to reach the highest number of 47.1 nm in result of 0.1 µM Cu^2+^ addition. Form 0.1 to 1.0 µM Cu^2+^ addition, the size of CQDs relatively stood at the same level somehow, with a moderate fluctuation between 49.5 and 52.0 nm. Interestingly, the upward trend of CQDs size increase (which attributes to the addition of 0.005–0.1 µM Cu^2+^ addition) might be related to CQD-Cu-CQD complexes making the size of CQDs increase. In Cu^2+^ concentration range from 0.1 to 1.0 µM, the possibility of the formation of these types of complexes decreases. However, a tremendous decrease in the size of CQDs was observed after addition of 1–10.0 µM of cupric ions (Fig. 10A), which the CQDs size dropped from 52.1 to 20.8 nm after addition of 10.0 µM Cu^2+^. This decreasing trend might be attributed to the different complexation mode between CQDs and cupric ions (CQD-ion). In order to further look at the mechanism of interaction between CQDs and these ions, zeta potential have also been investigated. The ζ-potential for CQDs was seen to be − 11.0 mV, which the negative charge started to increase after addition of 0.005 µM Cu^2+^, and experienced an astonishing escalation, nearly doubling till the concentration of Cu^2+^ gained 0.075 µM in the solution containing CQDs, to reach the highest amount (− 26.5 mV) at the 0.1 µM Cu^2+^ addition. When the concentration of Cu^2+^ varied between 0.25 and 1.0 µM, the ζ-potential for CQDs relatively remained at the same level, and fluctuated between − 26.8 and -27.9 mV. However, from 1.0 to 7.5 µM Cu^2+^ ions addition, the zeta potential significantly fell (− 8.5 mV), and continued to drop to reach the lowest number of − 1.0 mV by adding 10 µM Cu^2+^ (Fig. 10B).

It is worth suggesting that the increasing trend in the size of CQDs and ζ-potential after the addition of 0.005–0.1 µM Cu^2+^ may spot the possibility of electrostatic interactions between Cu^2+^ ions and CQDs^96^, leading to different complexation between Cu^2+^ ions and the functional groups located at the CQDs’ surface, which these types of complexations (CQD-ion-CQD) may increase the presence of electrons on their surface and finally make an enhancement in FL intensity of CQDs. Nonetheless, the addition of 1–10 µM cupric ions wrote another story. The size of CQDs remarkably decreases after the addition of 1.0–10.0 µM Cu^2+^, which can be attributed to the different complexation modes between CQDs and cupric ions (CQD-ion), which these types of complexations have also made the ζ-potential to approach the positive magnitudes. Actually, it means that Cu^2+^ ions may make complexes with all located heteroatoms on the surface of CQDs, and this makes their surface to become positive somehow, leading the size of CQDs and ζ-potential to decrease in this Cu^2+^ concentration addition range (1–10 µM). While the data may support the "turn-on" and "turn-off" mechanisms, additional theoretical studies using DFT have been conducted to study the complexation modes between CQDs and Cu^2+^ ions, the stability of these different complexation modes, and to clarify the proposed mechanisms behind our observations. In our theoretical studies, those metal ions (including Cu^2+^, Co^2+^, and Fe^2+^ ions) that have shown the highest effects on the FL emission of CQDs (Fig. 5B), have been used.

### Density functional theory (DFT)

**Figure 11:**
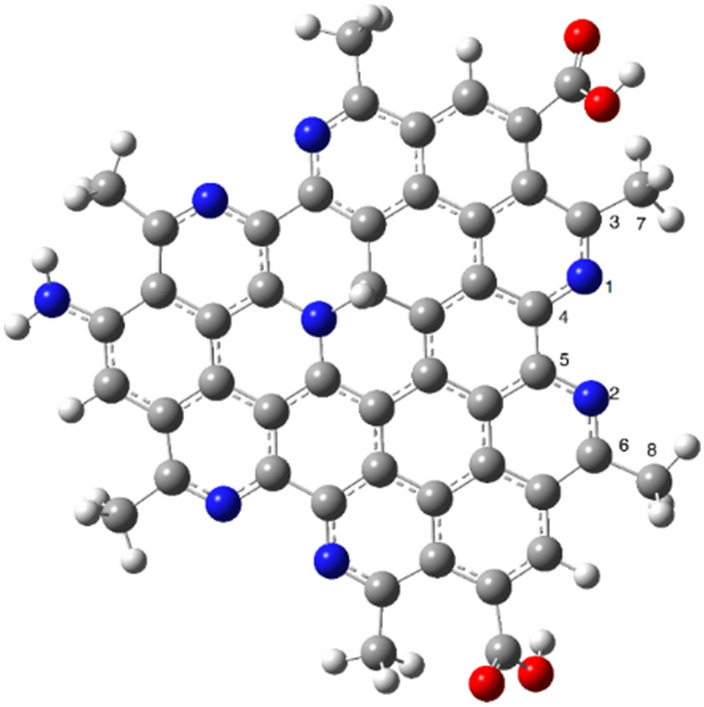
The optimized structure of the CQD at the BP86-D3/Def2-SVP level of theory.

The CQDs structure was optimized with and without metal ions at the BP86-D3/Def2-SVP level of theory. The proposed structure for the CQD has a flat geometry with specific bond lengths (N1-C4, C4-C5, N2-C5) around 1.36–1.45 Å (Table 3). The complexation between CQDs and metallic ions has significant effects on the geometrical parameters of the receptor (Fig. 11). For example, the length of N1-C4 and N2-C5 bonds increased from 1.36 up to 1.39 Å in the presence of Co^2+^, Cu^2+^, and Fe^2+^. On the other hand, metals are effective on the angles between N1-C4-C5, C3-N1-C4, and C7-C3-N1 atoms, in which Fe^2+^ and Cu^2+^ ions show more remarkable effects compared with Co^2+^. The calculated average metal-N bond length for the Co^2+^, Cu^2+^, and Fe^2+^ are 1.94, 1.98, and 1.85 Å, correspondingly. These results show that nitrogens of the pyridine-like rings play remarkable roles in complexion with metallic ions.

**Table 3:** The calculated geometrical parameters of the CQD complexes with different metals (the bond length and angles are in Å and degree, respectively).

Metal	N1-C4	C4-C5	N2-C5	C3-N1-C4	N1-C4-C5	C7-C3-N1	Metal-N
CQD	1.36	1.45	1.36	121.39	119.54	114.92	……
Co^2+^	1.39	1.43	1.38	119.92	118.93	117.52	1.94
Cu^2+^	1.38	1.44	1.37	121.19	120.22	117.67	1.98
Fe^2+^	1.37	1.40	1.38	120.54	115.40	112.66	1.85

**Figure 12:**
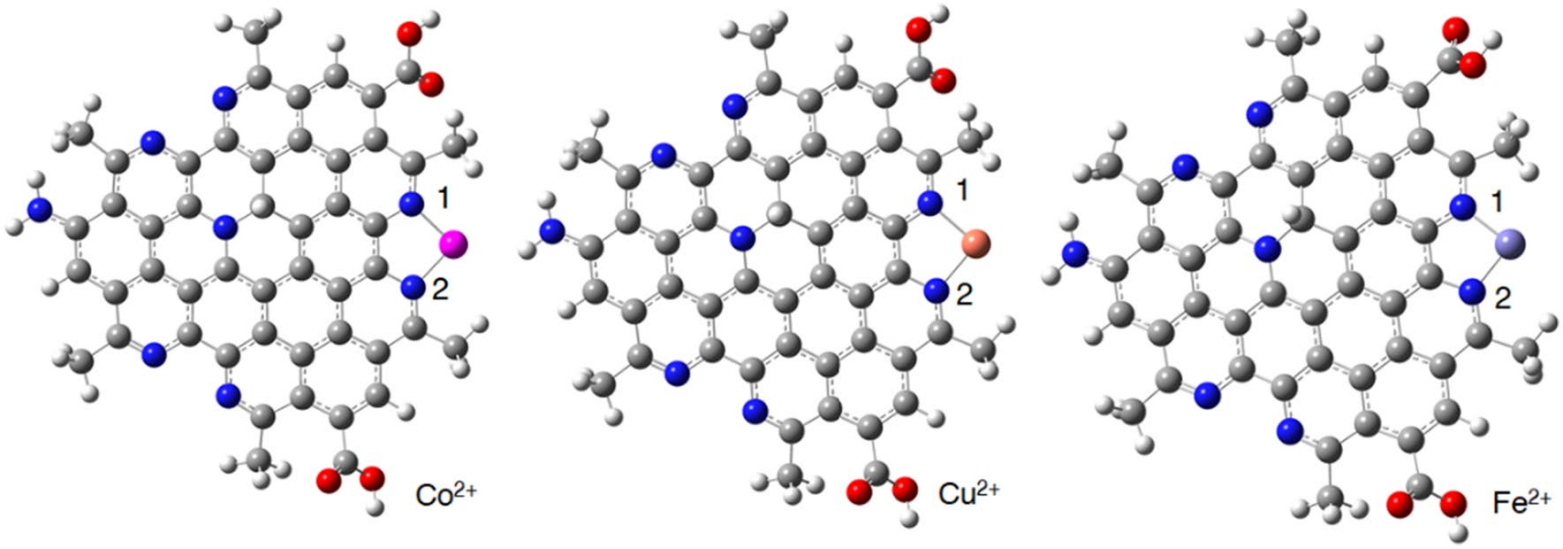
The optimized structures of CQD-ion (monomer) complexes at the BP86-D3/Def2-SVP level of theory.

**Figure 13:**
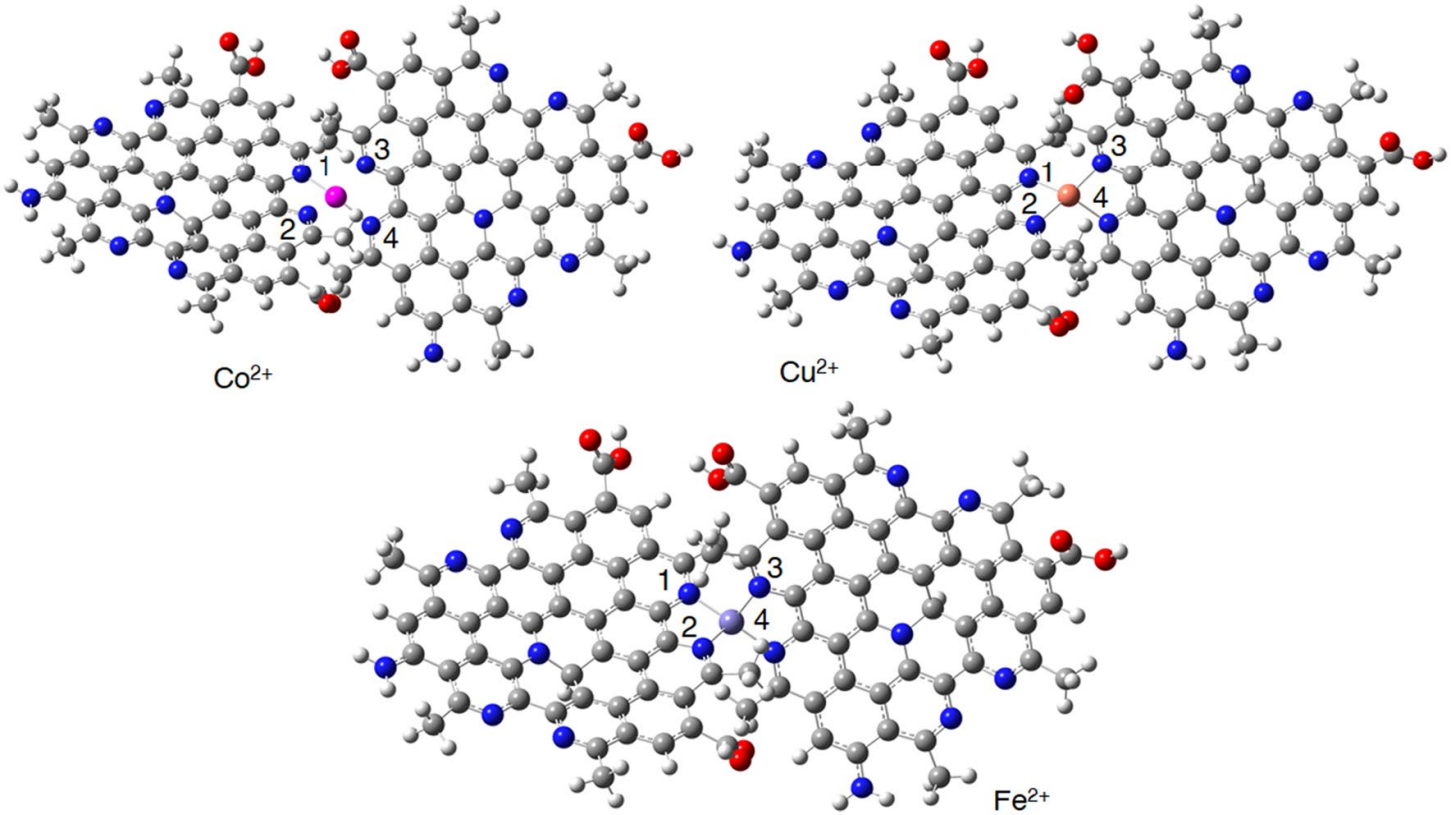
The optimized structures of CQD-ion-CQD (dimer) complexes at the BP86-D3/Def2-SVP level of theory.

The optimized structures of CQD-ion and CQD-ion-CQD complexes have shown in Figs. 12 and 13. The optimized structures of CQD-ion-CQD complexes indicate that these complexes have a tetragonal structures (Fig. 13). The dimerization process (CQD-ion-CQD) reduces the interactions between the metals and CQD, according to the calculated structural parameters. According to Table 4, the metal-N bond lengths increase in the presence of the second CQD.

**Table 4:** The calculated N-metal bond lengths (Å) of the CQD-ion-CQD complexes at the BP86-D3/Def2-SVP level of theory.

Metal	N1-Metal	N2-Metal	N3-Metal	N4-Metal
Co^2+^	1.95	1.98	1.99	1.96
Cu^2+^	2.04	2.05	2.05	2.05
Fe^2+^	1.90	1.94	1.94	1.89

The binding energy (Table 5) of the CQD complexes with different metals shows the stability of the corresponding structures, and the affinity of the CQD in selective complexation. By elevating the absolute value of binding energy, the stability of the complexes is improved. The calculated thermodynamic parameters indicate that CQD can form stable complexes with different metals. According to the calculated binding energies, the stability of the CQD-ion complexes is as follows: Fe^2+^ > Cu^2+^ > Co^2+^. Compared to CQD-ion, the dimer of CQD shows maximum stability in the presence of Cu^2+^. The higher stability of CQD-ion-CQD complex in the presence of Cu^2+^ can be in the same line with experimental observations.

**Table 5:** The calculated thermodynamic parameters (kcal.mol^–1^) for the CQD complexes with different metal ions.

	ΔE	ΔH	ΔG
Monomer			
Co^2+^	− 389.09	− 389.68	− 380.82
Cu^2+^	− 426.86	− 427.45	− 418.40
Fe^2+^	− 491.98	− 492.57	− 477.37
Dimer			
Co^2+^	− 728.90	− 730.09	− 693.91
Cu^2+^	− 751.26	− 752.45	− 718.16
Fe^2+^	− 750.53	− 751.71	− 715.31

The negative values of ΔH reveal that the complexation process is exothermic, and CQD-ion-CQD structures are much more stable than the CQD-ion complexes. The calculated Gibbs binding energies indicate that the complexation process is favorable from the thermodynamic viewpoint (ΔG < 0), revealing that the monomer and dimer of the CQD can form the most stable complexes with Fe^2+^ and Cu^2+^, respectively, which this result can be evidence for the experimental observation. Based on the experimental results, the synthesized CQD shows greater sensitivity against Cu^2+^ in low concentrations. The obtained theoretical results show that the CQD-ion-CQD complexes are more stable than CQD-ion structures. By increasing the concentration of the Cu^2+^, all the possible sites of the CQD for interaction with the metal ion will be occupied, reducing the possibility of the dimerization process. In other words, the dimerization process does not occur in the presence of a high concentration of Cu^2+^ because cupric ions make complexes with all the other possible sites (at higher concentrations) with lower stability, which agrees with the experimental observations. These complexations may suppress electron- or charge-transfer, and Turn-off the FL emission of CQDs.

It is possible to describe the interaction mechanism of metal ions and CQD through the frontier molecular orbital theory (Table 6). The complexation process has considerable effects on the electronic properties of the CQD. According to the results, the energy values of HOMO and LUMO orbitals of the CQD increased in the presence of the metal ions. The Co^2+^ and Fe^2+^ have considerable effects on the E_HOMO_ and E_LUMO_ of the CQD, respectively. The band gap (η) value shows the reactivity and conductivity of the CQDs against the metal ions. According to Table 6, the synthesized CQD possess the maximum band gap or chemical hardness in complexation with Cu^2+^. This result confirmed that the CQDs bear a remarkable sensitivity relative to Cu^2+^. The larger absolute values of electronic chemical potential are associated with greater chemical reactivity. The calculated chemical potential values (μ) for both the CQD-ion and CQD-ion-CQD complexes with Cu^2+^ have the minimum reactivity than the corresponding complexes, which lower reactivity showing more excellent stability for the CQD complex with the Cu^2+^ ion. These remarkable changes in the electronic properties of CQD in the presence of Cu^2+^ confirmed that CQDs have a higher sensitivity toward Cu^2+^ than Co^2+^ and Fe^2+^ ions.

**Table 6:** The calculated quantum reactivity indices (eV) of the CQD and its complexes with different metal ions.

	E_HOMO_	E_LUMO_	η	μ
Monomer				
Co^2+^	− 9.85	− 9.37	0.48	− 9.61
Cu^2+^	− 9.64	− 8.38	1.26	− 9.01
Fe^2+^	− 9.43	− 8.93	0.50	− 9.18
CQD	− 4.68	− 3.36	1.32	− 4.02
Dimer				
Co^2+^	− 8.23	− 7.94	0.29	− 8.09
Cu^2+^	− 8.27	− 6.96	1.31	− 7.62
Fe^2+^	− 8.19	− 7.86	0.33	− 8.03

**Figure 14:**
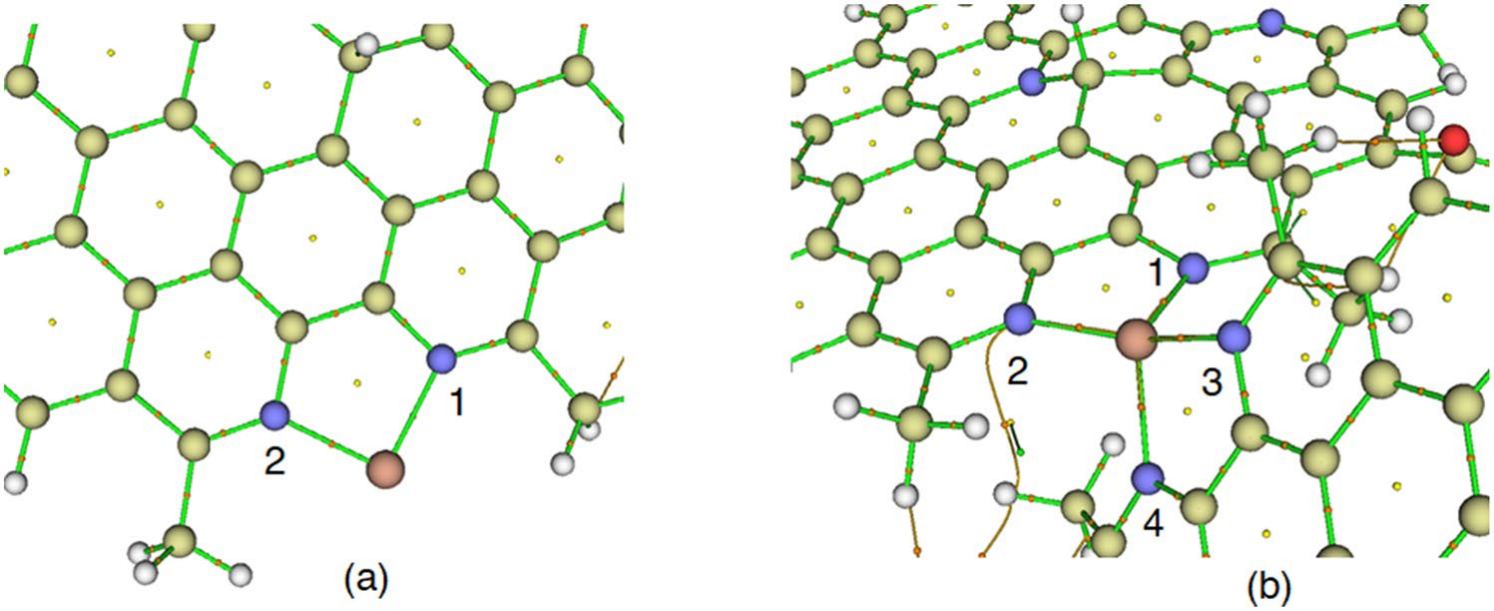
A schematic presentation of molecular graph for the CQD-ion (**a**) and CQD-ion-CQD (**b**) complexes with the metals.

To compare the reactivity of the CQD against the corresponding metal ions, QTAIM analysis was performed to calculate the topological parameters at the bond critical points (BCPs). Figure 14 shows the molecular graph of the QCD complexes with the metals. This figure confirms that the CQDs have shown remarkable interactions with the corresponding metals, which shows the complex formation process. According to this figure, the metals between two CQDs play as a bridge that can increase the charge and electron transfer between the CQD, which possibly can have significant effects on the spectroscopic features of the CQD.

The value of the electron density, ρ(r), at the BCP, shows the strength of the interactions. The calculated ρ(r) values for the N-Cu^2+^ are greater than those of N-Co^2+^ and N-Fe^2+^, which confirms that Cu^2+^ has more interaction with the CQD than the other two metal ions (Table 7). On the other hand, the calculated values of the electron density for the Cu^2+^ with the CQD are much more than the Co^2+^ and Fe^2+^ structures, confirming more reactivity for the CQD. The positive values of the Laplacian, ∇^2^ρ(r), show that the N-metal bonds are partially covalent (covalent/electrostatic) in nature. The ratio of the kinetic energy density (G) to the potential energy density (V) can be employed to determine and investigate the nature of the interaction from the non-covalent or covalent viewpoint. The calculated − G/V values are between 0.5 and 1.0, which confirms that the nature of the interactions between metals and the CQD is partially covalent. Moreover, the obtained values for the Cu^2+^ bonds are smaller than the corresponding values for other complexes, indicating that Cu^2+^ has stronger interactions with the N atoms of the CQD than the Co^2+^ and Fe^2+^ metals, which might be a piece of crystal evidence for possible dimerization in experimental observations.

**Table 7:** The calculated topological parameters (au) at the BCP for the CQD complexes with different ions.

	Bond	ρ	$\nabla$ ^2^ρ(r)	− G/V	ELF	LOL
Monomer						
Co^2+^	1	0.1022	0.4901	0.8426	0.1599	0.2993
	2	0.0987	0.4800	0.8475	0.1536	0.2939
Cu^2+^	1	0.0926	0.3715	0.8089	0.1670	0.3093
	2	0.0930	0.3719	0.8078	0.1681	0.3101
Fe^2+^	1	0.0928	0.5108	0.9113	0.1412	0.2891
	2	0.0986	0.4651	0.8912	0.1498	0.2872
Dimer						
Co^2+^	1	0.0969	0.4276	0.8364	0.1652	0.3065
	2	0.0929	0.3863	0.8312	0.1725	0.3114
	3	0.0937	0.4338	0.8385	0.1620	0.3040
	4	0.0919	0.3773	0.8304	0.1732	0.3120
Cu^2+^	1	0.1156	0.4965	0.8351	0.2065	0.3378
	2	0.1014	0.4709	0.8543	0.1659	0.3085
	3	0.1007	0.4560	0.8500	0.1698	0.3114
	4	0.1145	0.4769	0.8307	0.2114	0.3411
Fe^2+^	1	0.0971	0.4100	0.8417	0.1611	0.2982
	2	0.0918	0.3912	0.8513	0.1692	0.3001
	3	0.0921	0.4231	0.8611	0.1591	0.3242
	4	0.0919	0.3712	0.8514	0.1701	0.3027

**Figure 15:**
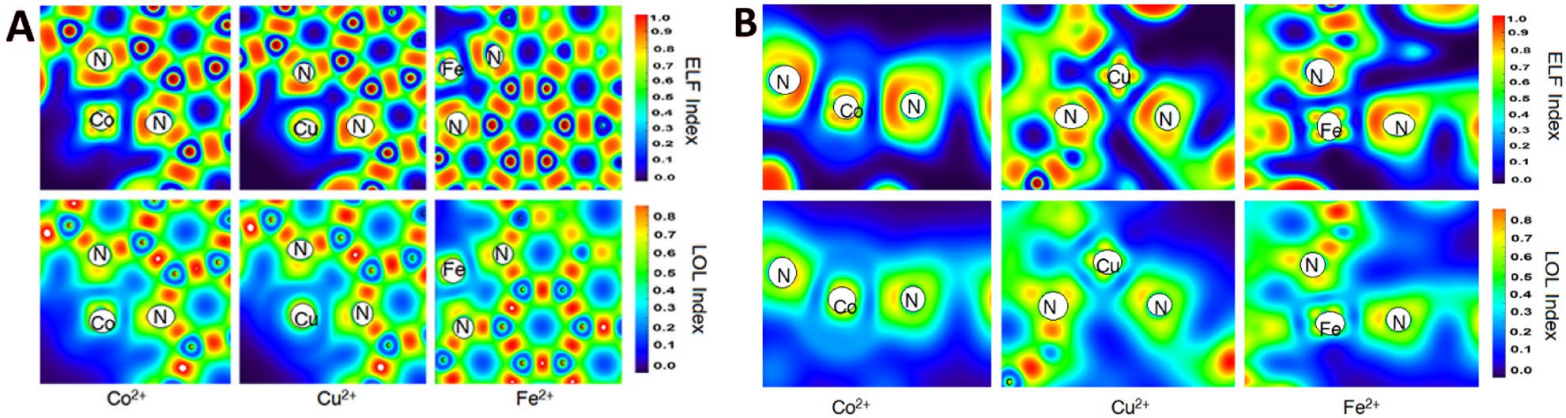
The ELF and LOL plots of (**A**) CQD-ion complexes and (**B**) CQD-ion-CQD complexes with different metal ions.

The chemical definition of the ELF is similar to the LOL, in which LOL indicates the gradients of the localized orbitals, and ELF reveals the electron pair density. The calculated ELF and LOL values confirm that the Cu^2+^ metal interacts more with the N atoms of the CQD than other metals. According to Fig. 15, the obtained ELF plots show that Cu^2+^ metal forms stronger bonds with both N atoms of the CQD, while the bonds of other metals are partially electrostatic in nature. Moreover, the ELF and LOL plots reveal that the Cu^2+^ ions possess stronger interactions with the π orbitals of the C=C and bonds of the CQD than the other metals. Overall, quantum chemistry calculations indicated that the CQDs have different structural properties in the presence of the metal ions, greater reactivity, and interactions with the Cu^2+^ due to the stronger molecular orbital interactions between Cu^2+^ metal and the CQDs. In fact, Cu^2+^ ions can act as a bridge between two CQDs, and make CQD-ion-CQD complexes, which can be in line with DLS results in which CQD’s size increased in the 0.005–0.1 µM addition of Cu^2+^ ions because of CQD-Cu^2+^-CQD complexes. Thus, this dimerization process can, in turn, increase charge and electron transfer, and it can have a substantial effect on the spectroscopic properties of CQDs. This might back up the proposed mechanism for the Turn-on state. In other words, by increasing the concentration of the Cu^2+^, all the possible sites of the CQDs for interaction with the metal ion will be occupied, and the dimerization process (CQD-Cu^2+^-CQD) does not occur because cupric ions make complexes with all the other possible sites with lower stability, which might agree with the observed decrease in the size of CQDs after addition of 1–10.0 µM of Cu^2+^ ions. These complexation modes (CQD-ion) may decrease the electron and charge transfer, and quench the FL emission of CQDs. The observed decreasing trend in the zeta potential and the size of CQDs after 1–10.0 µM addition of Cu^2+^ ions might also back up the CQD-ion complexation mode in the micro-molar addition range, and can be a strong evidence for the CQD’s FL emission signal intensity decrease.

## Conclusion

The presence of Cu^2+^ is closely associated with the development and progression of Wilson's disease (WD). Accurate measurement of copper levels is crucial for early diagnosis of WD in clinical settings. This study developed CQDs as a new fluorescence-based sensing platform to detect Cu^2+^ in aqueous solutions and serum samples of healthy and affected individuals by WD. The successful preparation of CQDs, which contain strong adsorption sites such as carbonyl, hydroxyl, and amide functional groups, as well as pyridine-like rings, was confirmed by FT-IR and XPS analyses. The amorphous graphitic sp^2^ carbon building blocks of CQDs were approved through HR-TEM. The atomic percentage and the uniform spatial distribution of CQDs can be seen from elemental mapping and EDS analysis. The CQDs also showed a strong blue fluorescence emission (wavelength ∼375 nm) under UV light with an excitation wavelength of 300 nm. Interestingly, adsorption of Cu(II) on the CQDs’ surface through complexation with the surficial functional groups leads to CQDs’ FL emission enhancement (Turn-on state) at the nano-molar level on one hand, and FL emission quenching (Turn-off state) at the micro-molar addition of Cu(II), on the other hand. The sensor showed high selectivity and sensitivity for Cu(II) detection with a LOD of 0.001 µM, and a dynamic range of 0.001–10 µM. Moreover, a paper-based test for efficient Cu(II) monitoring illustrates an obvious color change under a 365 nm UV lamp.

According to the mechanism of our observations, according to DLS results, the size of CQDs experienced a significant increase after the addition of 0.005–0.1 µM Cu^2+^ ions, which might be an evidence for CQD-ion-CQD complexation mode in the nano-molar addition of Cu^2+^. However, the size of CQDs remarkably decreased when the amount of copper ions reached 1–10.0 µM in the solution containing CQDs, which clearly highlights a change in the complexation modes between CQDs and Cu^2+^ ions (CQD-ion). Zeta potential also showed an increase in nanomolar addition range of the analyte, and however, a decrease in the micromolar addition range of the analyte, which is in agreement with the results of DLS, and both together confirm the changes in the modes of complexations between CQDs and Cu^2+^ ions, based on different concentration ranges of copper ions.

Hence, these different complexation modes could significantly change the FL emission intensity of the CQDs, and cause two distinctive phenomena. Furthermore, the DFT calculations revealed that CQDs bear strong interactions with the Cu^2+^, and mainly, Cu^2+^ made stronger binding energies with CQDs. The nature of interaction between CQDs and metal ions was also explored, which shows the high sensitivity of our sensor toward Cu^2+^ ions. Charge analysis also delineated that the Cu^2+^ can act as a bridge, and get adsorbed between two CQDs (CQD-ion-CQD) via complexation with the N atoms of pyridine-like rings located at the CQDs’ surface, which can increase the charge and electron transfer between the CQDs, and affects the spectroscopic features of the CQD. This result is absolutely in line with the increase in the size and zeta potential of CQDs when 0.005–0.1 µM Cu^2+^ ions are added to the solution containing CQDs. It means that if the concentration of CQDs is at nanomolar range, Cu^2+^ ions possibly make CQD-ion-CQD complexation mode, which these types of complexations can increase the electron and charge transfer, and possibly, increase the FL emission intensity. The electron density values for the N-Cu^2+^ are greater than those of N-Co^2+^ and N-Fe^2+^, which confirms the selectivity of CQDs toward Cu^2+^, and also higher tendency of CQDs to make complex with Cu^2+^ via N–pyridinic rings at their surface (CQD-ion-CQD). This observation was further approved by the HOMO–LUMO band gap (Eg) and Frontier molecular orbital analysis. However, when the concentration of copper ions increases, all of adsorption sites on the surface of CQDs might be occupied by these ions, and the complexation mode between CQDs and Cu^2+^ changes to CQD-ion mode at 1–10.0 µM Cu^2+^ ions addition. At this concentration range, the size and zeta potential decreases, which can be in agreement with DFT results, and approve the change in the complexation mode, and can decrease electron and charge transfer, and finally, decrease the CQDs FL emission intensity.

This is the first reported research to determine Cu^2+^ from nano-molar to micro-molar levels through two different complexation states causing two different phenomena. The sensor's potential for Cu^2+^ detection was tested in the serum samples of healthy and affected individuals by WD, and compared with ICP-OES results. The results showed a strong correlation between the measured Cu^2+^ in the plasma of affected individuals by WD using the proposed technique and by ICP-OES. To extend our knowledge, the CQD-based sensing system offers several advantages such as cost-effectiveness, linear response, rapid detection, high precision, convincing accuracy, high sensitivity, and excellent selectivity toward Cu^2+^ ions, making the sensor a promising diagnostic tool for monitoring the concentration of Cu^2+^ ions in clinical settings for the diagnosis of Wilson's disease.

### Supplementary Information

Supplementary Information.

## Data Availability

All the associated with this work are presented here (and its Supplementary Information file) and further will be made available on reasonable request. Correspondence and requests for materials should be addressed to A.R.
