# Supplementary material for: Selective and sensitive CQD-based sensing platform for Cu2+ detection in Wilson’s disease
Source: Sci Rep. 2024 Jun 8;14:13183. doi: 10.1038/s41598-024-63771-9 (PMC11162432; doi:10.1038/s41598-024-63771-9)
Supplement: Supplementary file 1 — Supplementary Information. [file 41598_2024_63771_MOESM1_ESM.docx]

**Supporting Information**

**Selective and Sensitive CQD-Based Sensing Platform for Cu^2+^ Detection in Wilson’s disease**

Armin Zarei ^a^, Aram Rezaei *^b^, Mohsen Shahlaei ^b^, Zhaleh Asani ^c^, Ali Ramazani*^a^, Chuanyi Wang*^d^

*^a^ Department of Chemistry, University of Zanjan, Zanjan 45371-38791, Iran.*

*^b^ Nano Drug Delivery Research Center, Health Technology Institute, Kermanshah University of Medical Sciences, Kermanshah, Iran.*

*^c^ Radiology Department, Kermanshah University of Medical Sciences, Kermanshah, Iran.*

*^d^ School of Environmental Science and Engineering, Shaanxi University of Science and Technology, Xi'an 710021, P. R. China.*

Corresponding authors: Aram Rezaei, Email: [aram.rezaei@gmail.com](mailto:aram.rezaei@gmail.com); Ali Ramazani, Email: [aliramazani@gmail.com](mailto:aliramazani@gmail.com); Chuanyi Wang, Email: wangchuanyi@sust.edu.cn.

**
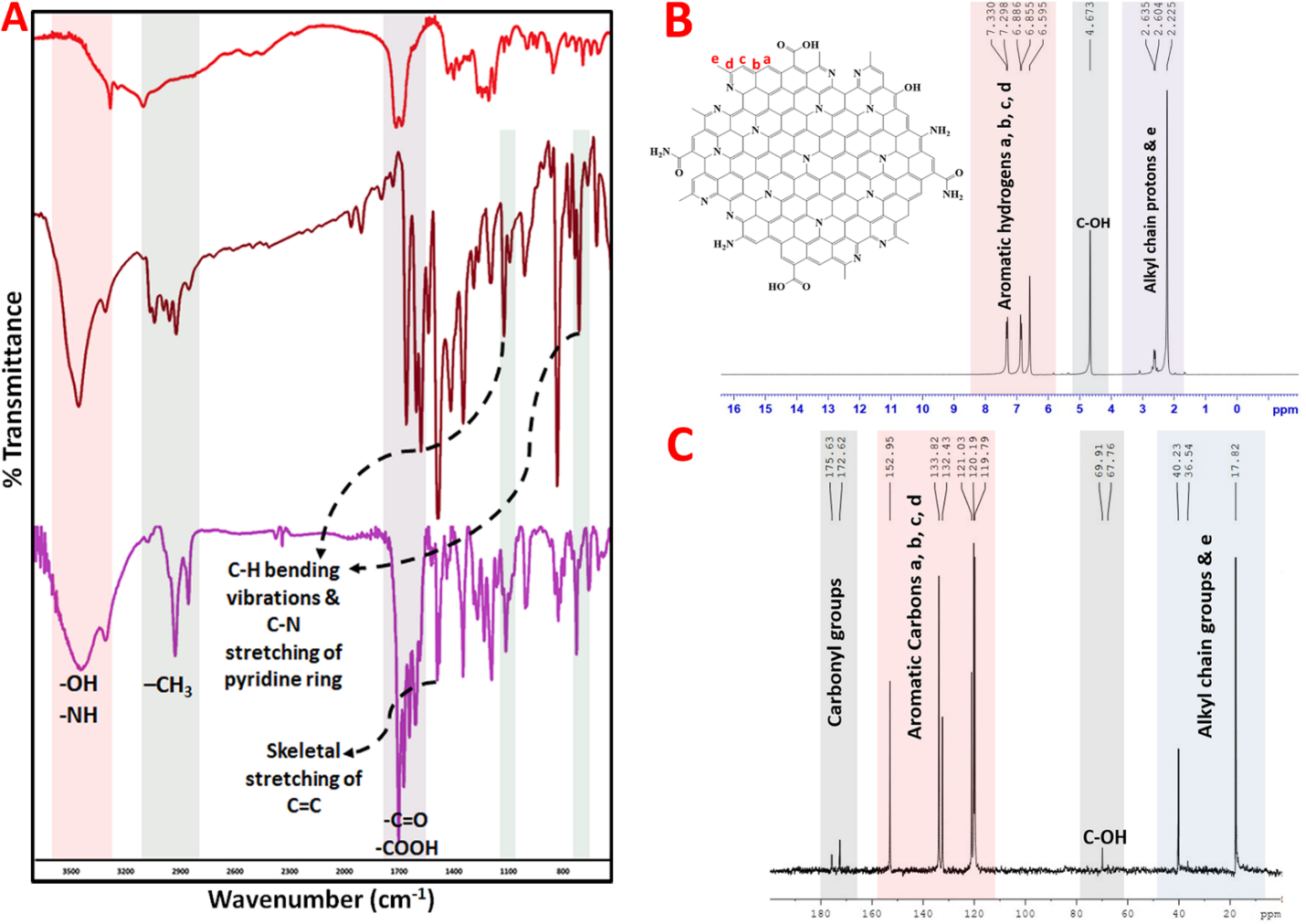
**

**Figure S1.** (A) FT-IR spectrum of Citric acid (Red), Neocuproine (crimson), and CQDs (Purple), (B) ^1^H NMR and (C) ^13^C NMR spectra of CQDs.

**Table S1.** Summary of human serum samples in the current study.

|  | **Male** | **Female** |
| --- | --- | --- |
| **Human control group (n = 1)** | | |
| Number of donors | 1 (100%) | - |
| Age (median, IOR) | 36 |  |
| **WD Patients (n = 1)** | | |
| Number of donors | 1 (100%) | - |
| Age (median, IOR) | 29 | - |

**
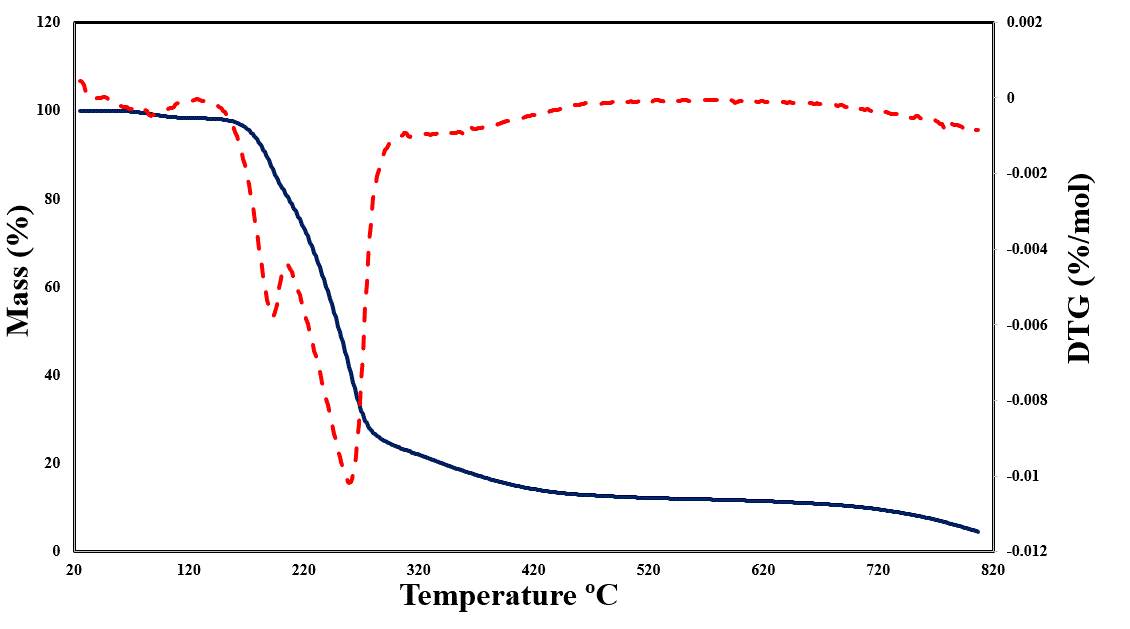
**

**Figure S2.** TGA and DTG curves for the CQDs.


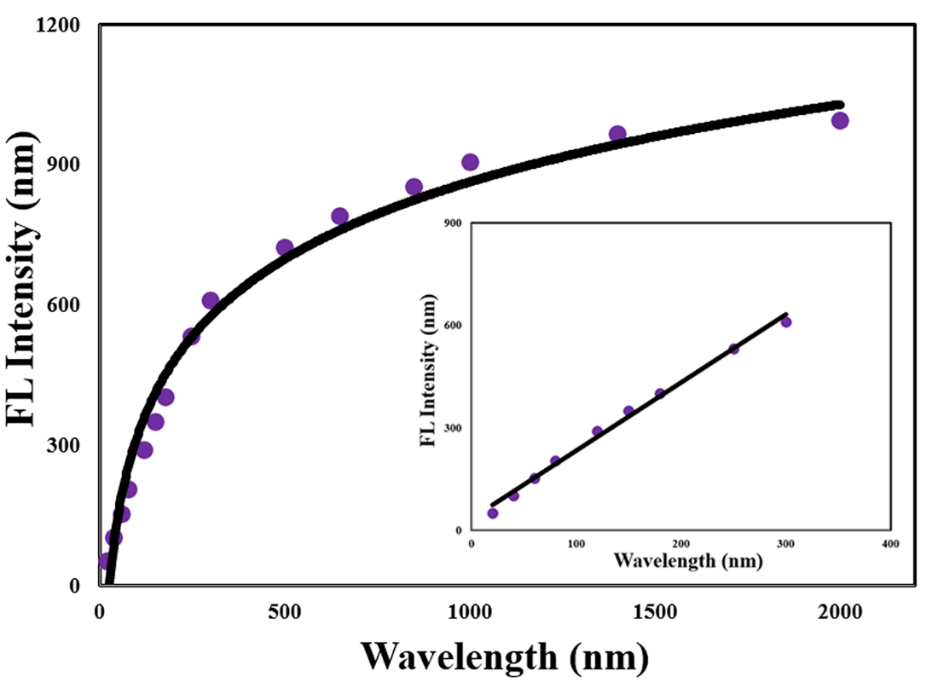


**Figure S3.** Concentration-dependent FL response of CQDs in pH 7.0 PBS. The inset shows a linear correlation between FL intensity and CQD concentration in the range of 0 to 300 ng mL-1 (violet dots).


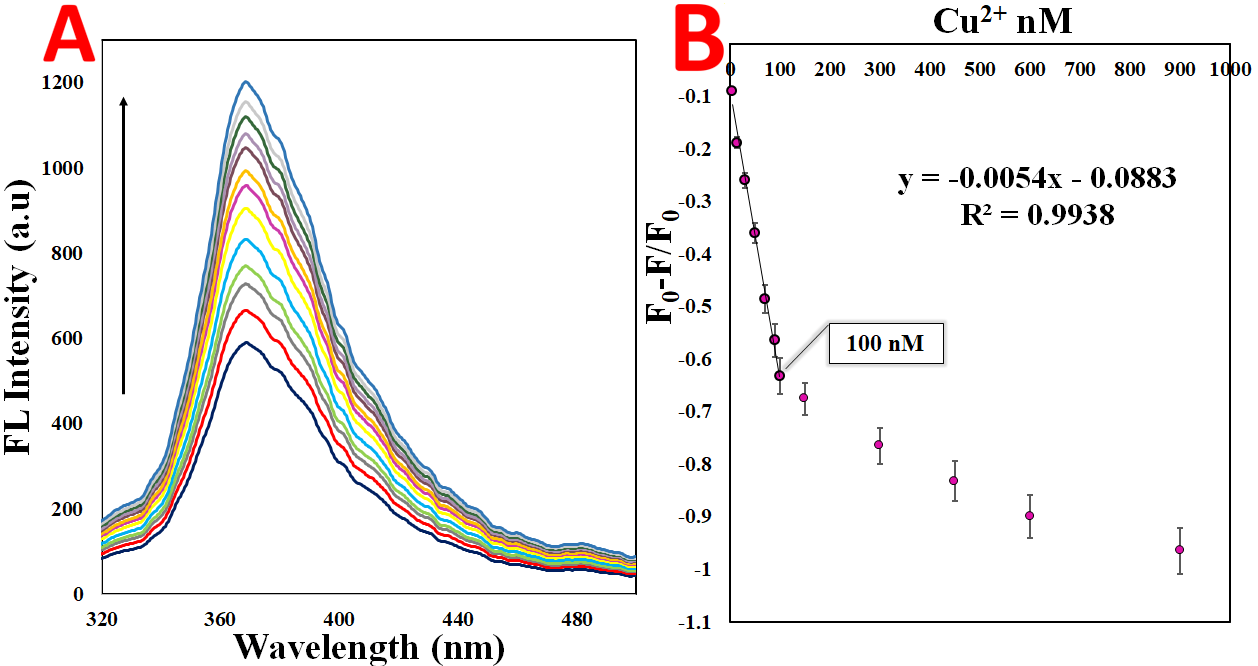


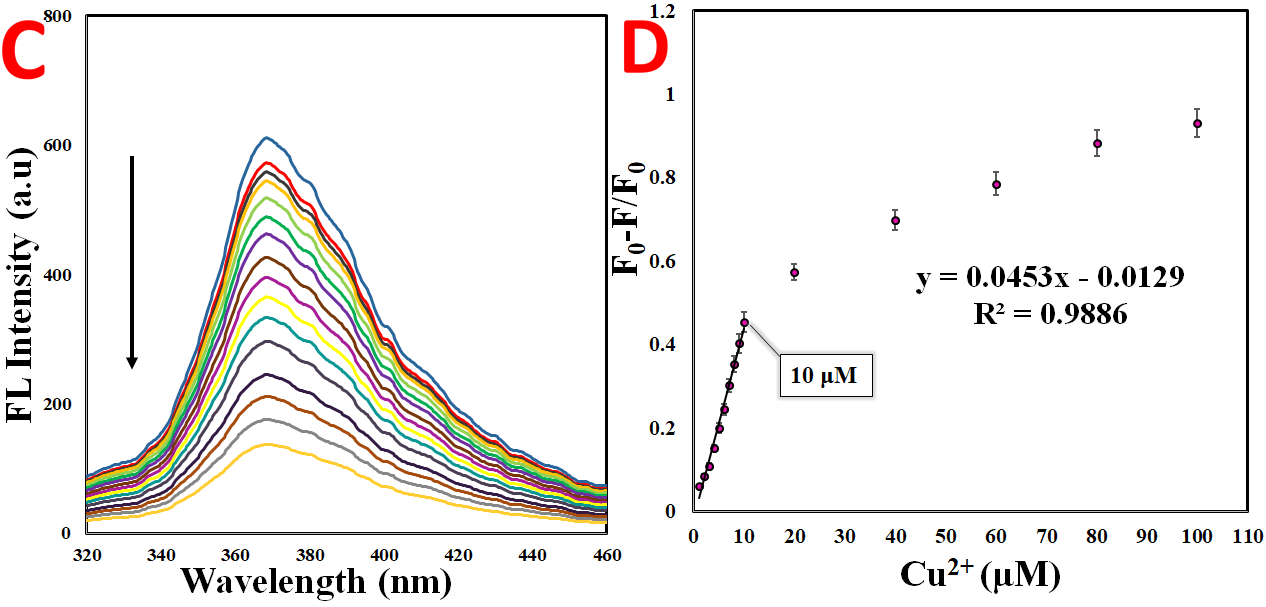


**Figure S4.** FL response of CQDs (A, B) after addition of 0, 0.005, 0.015, 0.03, 0.05, 0.07, 0.09, 0.1, 0.15, 0.3, 0.45, 0.6, 0.9 µM; and (C, D) after addition of 0, 1, 2, 3, 4, 5, 6, 7, 8, 9, 10, 20, 40, 60, 80, 100 µM Cu^2+^ (violet dots) in pH 7.0 PBS. Inset illustrate the plot of F_0_-F/F_0_ of CQDs (300 ng mL^-1^) vs the Cu^2+^ concentration (with a linear response from 0.005-0.1 µM for ‘’Turn-on’’ and from 1-10 µM cu^2+^ ions for ‘’Turn-off’’ states, correspondingly).


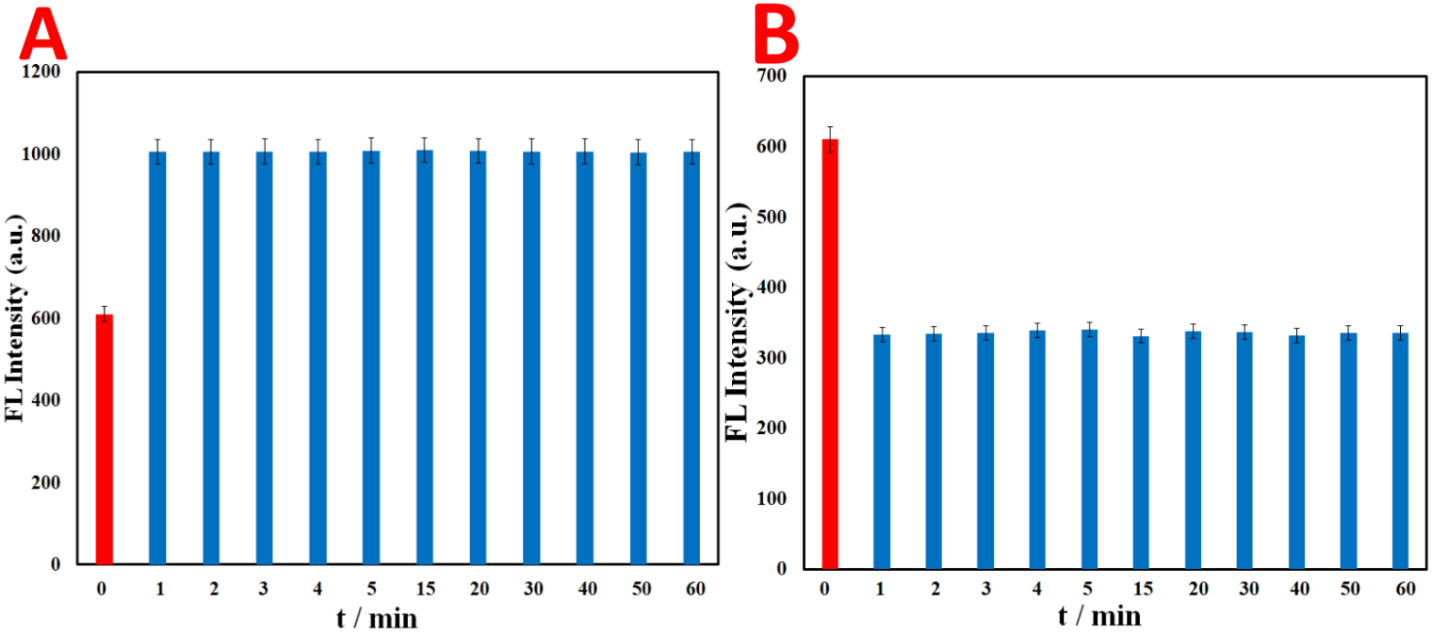


**Figure S5.** Time-dependent FL response of the 300 ng mL^-1^ CQDs in pH 7.0 PBS to A) the addition of 0.1 µM and B) 10 µM copper ions.


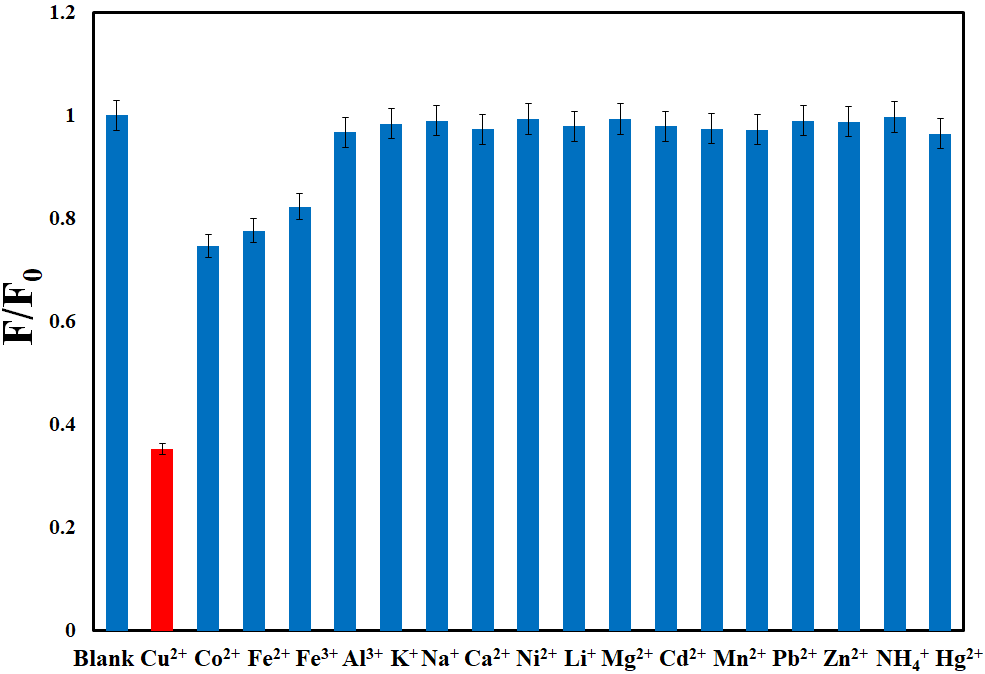


**Figure S6.** Selectivity of the CQDs-based probe for Cu^2+^ ions over other ions in pH 5.0 PBS
solution: concentrations of the CQDs and metallic ions were 300 ng mL^-1^ and 10 µM, correspondingly.

**Table S2.** Determination of Cu^2+^ content in 10% deproteinized human plasma samples. (Linear range of µM addition of cupric ions-quenching trend).

| **Samples** | **Cu^2+^ Spiked (µM)** | **Cu^2+^ Measured (µM)** | **Recovery (%)** | **RSD (%)** |
| --- | --- | --- | --- | --- |
| 10% Plasma | 0 | 0.179 ± 0.0096 | **-** | 5.4 |
| 10% Plasma + Cu^2+^ | 4 | 4.138 ± 0.135 | 103.4 | 3.2 |
| 10% Plasma + Cu^2+^ | 8 | 7.985 ± 0.130 | 99.8 | 1.63 |
| 10% Plasma + Cu^2+^ | 10 | 10.149 ± 0.188 | 101.5 | 1.85 |


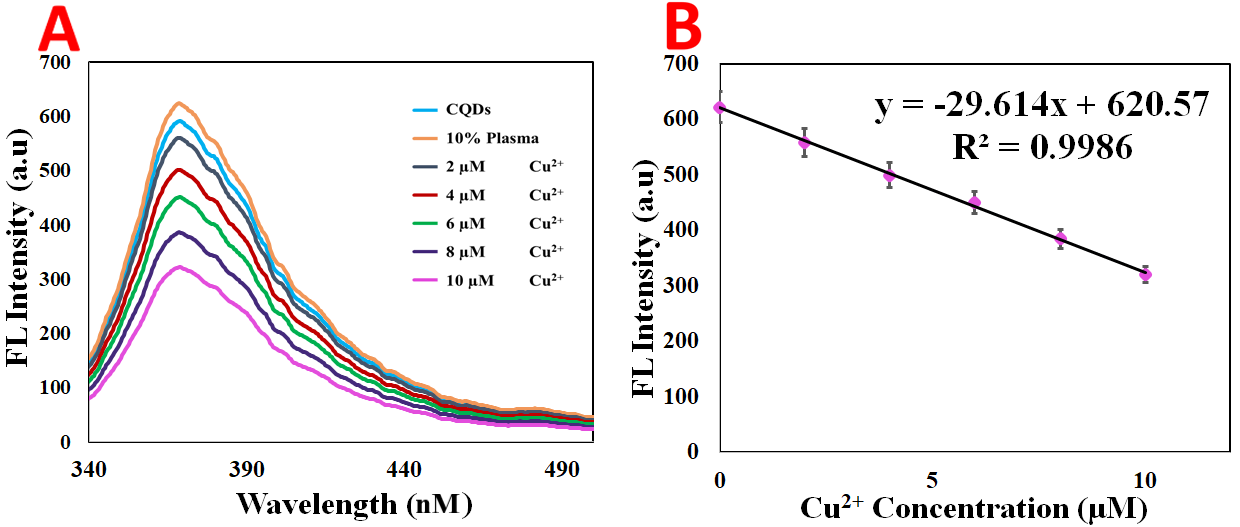


**Figure S7.** Sensing of Cu^2+^ in human plasma sample using the CQD-based system. (A) FL spectra (λ_ex_ = 300 nm) of CQDs (300 ng mL^-1^ μM) upon addition of cupric ions (2-10 µM) to 10% deproteinized plasma diluted with pH 7.0 PBS buffer; (B) Calibration curve of CQD-based sensor (300 ng mL^-1^) µM at pH 7.0 PBS (PBS buffer, 50 mM) revealing a linear correlation between FL intensity (λ_ex_ = 300 nm, λ_em_ = 375 nm) and Cu^2+^ concentration (2-10 µM). (The emission spectra and calibration curve were taken 4 min after mixing).
